# *Leishmania* proteophosphoglycans regurgitated from infected sand flies accelerate dermal wound repair and exacerbate leishmaniasis via insulin-like growth factor 1-dependent signalling

**DOI:** 10.1371/journal.ppat.1006794

**Published:** 2018-01-19

**Authors:** Emilie Giraud, Tereza Lestinova, Tamsyn Derrick, Oihane Martin, Rod J. Dillon, Petr Volf, Ingrid Műller, Paul A. Bates, Matthew E. Rogers

**Affiliations:** 1 Department of Immunology and Infection, Faculty of Infectious Tropical Diseases, London School of Hygiene and Tropical Medicine, London, United Kingdom; 2 Department of Parasitology, Faculty of Science, Charles University, Prague, Czech Republic; 3 Department of Clinical Research, Faculty of Infectious Tropical Diseases, London School of Hygiene and Tropical Medicine, London, United Kingdom; 4 Department of Disease Control, London School of Hygiene and Tropical Medicine, London, United Kingdom; 5 Division of Biomedical and Life Sciences, Lancaster University, Lancaster, United Kingdom; 6 Department of Medicine, Section of Immunology, Imperial College London, St Mary’s Campus, London, United Kingdom; Washington University School of Medicine, UNITED STATES

## Abstract

*Leishmania* parasites are transmitted to vertebrate hosts by female phlebotomine sand flies as they bloodfeed by lacerating the upper capillaries of the dermis with their barbed mouthparts. In the sand fly midgut secreted proteophosphoglycans from *Leishmania* form a biological plug known as the promastigote secretory gel (PSG), which blocks the gut and facilitates the regurgitation of infective parasites. The interaction between the wound created by the sand fly bite and PSG is not known. Here we nanoinjected a sand fly egested dose of PSG into BALB/c mouse skin that lead to the differential expression of 7,907 transcripts. These transcripts were transiently up-regulated during the first 6 hours post-wound and enriched for pathways involved in inflammation, cell proliferation, fibrosis, epithelial cell differentiation and wound remodelling. We found that PSG significantly accelerated wound healing *in vitro* and in mice; which was associated with an early up-regulation of transcripts involved in inflammation (IL-1β, IL-6, IL-10, TNFα) and inflammatory cell recruitment (CCL2, CCL3, CCL4, CXCL2), followed 6 days later by enhanced expression of transcripts associated with epithelial cell proliferation, fibroplasia and fibrosis (FGFR2, EGF, EGFR, IGF1). Dermal expression of IGF1 was enhanced following an infected sand fly bite and was acutely responsive to the deposition of PSG but not the inoculation of parasites or sand fly saliva. Antibody blockade of IGF1 ablated the gel’s ability to promote wound closure in mouse ears and significantly reduced the virulence of *Leishmania mexicana* infection delivered by an individual sand fly bite. Dermal macrophages recruited to air-pouches on the backs of mice revealed that IGF1 was pivotal to the PSG’s ability to promote macrophage alternative activation and *Leishmania* infection. Our data demonstrate that through the regurgitation of PSG *Leishmania* exploit the wound healing response of the host to the vector bite by promoting the action of IGF1 to drive the alternative activation of macrophages.

## Author summary

Female phlebotomine sand flies efficiently transmit *Leishmania* parasites, yet the underlying mechanisms remain poorly understood. During transmission, promastigote secretory gel (PSG) regurgitated from the blocked sand fly gut promote *Leishmania* infection and exacerbates disease. Here we investigate mouse ear-skin response to PSG and find that a significant proportion of up-regulated transcripts are involved in wound healing. Following a wound to skin preconditioned with PSG, key transcripts associated with inflammation and cell recruitment were enhanced by 4 hours and those associated with epithelial cell differentiation, proliferation and fibrosis were enhanced in the late phase of wound healing; including insulin growth factor-1 (IGF1) and its receptor. The presence of PSG significantly accelerated wound closure *in vitro* and *in vivo*, and this was directed by the presence of IGF1. IGF1 was essential to both the wound healing and disease-exacerbating properties of PSG, arising from its ability to alternatively activate macrophages. IGF1-blockade efficiently reduced the virulence of *Leishmania* infection from sand fly bite transmission indicating that it is essential to the function of PSG during natural infection. Our findings open the dual possibility of exploiting PSG, or defined components thereof, as a novel wound healing therapy and provide new targets for anti-leishmanial therapeutic design.

## Introduction

*Leishmania* is a genus of protozoan parasites transmitted by the bites of infected phlebotomine sand flies. Infection by sand fly bite is very efficient and difficult to replicate using needles. Along with the parasites, the sand fly injects saliva and a parasite-derived, glycan-rich gel termed promastigote secretory gel (PSG)–both of which have been shown to enhance cutaneous leishmaniasis in mice [1–6]. This is in part due to this mode of transmission, which has a significant influence on the virulence of the resulting infection. Despite this, we know little of the early immune events that follow transmission.

In the sand fly midgut *Leishmania* promastigotes develop into infectious metacyclic promastigotes stages. This process of metacyclogenesis is accompanied by the accumulation of PSG [7–10]. In mature infections, the PSG can block the anterior sand fly midgut [9]. This impairs the ability of the fly to bloodfeed and modifies the bloodfeeding behaviour of the sand fly, resulting in more transmission attempts (the ‘blocked fly hypothesis’ of *Leishmania* transmission) [9,10]. In addition, the PSG influences the mode of parasite transmission by promoting regurgitation of higher numbers of parasites per bite than would be delivered by inoculation of those in the proboscis alone [6,11]. PSG proved to be intimately involved in *Leishmania* transmission when co-infection experiments revealed that it potently exacerbated disease in cutaneous and visceral models of leishmaniasis [6,12,13]. The component responsible for the *Leishmania*-exacerbating properties of PSG is filamentous proteophosphoglycan (fPPG), the largest molecule secreted by *Leishmania* promastigotes inside the sand fly gut [6,14]. PSG promotes both macrophage recruitment and infection [12].

Depending on the balance of Th1 or Th2 cytokines in the tissue microenvironment macrophages may be activated in one of two directions. Classically activated macrophages (CAMΦ) arise from exposure to Th1 cytokines or mediators such as interferon gamma (IFN-γ), tumour necrosis factor alpha (TNFα) and IL-12, and express high levels of inducible nitric oxide synthase (iNOS/NOS2) that catabolises L-arginine into the *Leishmania*-toxic metabolite nitric oxide (NO). Such macrophages have a M1 phenotype. In skin iNOS is induced in the early phase of infection or wound healing [15,16] when macrophages respond to innate recognition pathways and the activation of Toll-like receptors (TLRs) [17,18]. In contrast, macrophages that express high levels of arginase1 (Arg1) in response to Th2 type cytokines such as IL-4, IL-10, IL-13, IL-21 and IL-33 hydrolyse L-arginine into L-ornithine, the precursor for polyamines; directing L-arginine away from iNOS and further suppressing the production of NO. As a result, alternatively activated (M2) macrophages (AAMΦ) are associated with resistance to helminth infections, allergic conditions such as asthma and Th2-induced pathologies [19,20]. Leishmaniasis is intimately linked with host arginase since polyamines are necessary intracellular nutrients and chronically high arginase activity is associated with uncontrolled parasite growth and cutaneous pathology in mice [21,22]. Furthermore, high arginase levels have been strongly associated with active cutaneous and visceral leishmaniasis in humans [23–26]. Previously, we found that fPPG from PSG attracted macrophages to the site of injection, promoted their alternative activation and increased their level of arginase activity; resulting in enhanced parasite survival and growth in mouse skin [12].

During the first few days after their inoculation *Leishmania* parasites are at their most vulnerable to the potent innate immune responses of the host to the sand fly bite [27]. This is exaggerated for sand flies since they, in proportion to their small size, inflict significant damage to the skin—a female sand fly lacerates the capillary loops of the upper dermis with its serrated proboscis to feed from the resulting pool of blood. This means that the PSG operates in a wound environment. In that regard it was found that resistance of mice to *Leishmania major* infection was associated with high collagen deposition and a faster rate of wound healing, controlled in part by three leishmaniasis susceptibility loci mapped to chromosome 17 [28]. However, it has also been established experimentally in mice that *Leishmania amazonensis* can readily metastasize to a fresh cut in the skin [29], and clinical cases of new cutaneous lesions appearing following local trauma to the skin have been recorded [30 and references therein]. Therefore, our understanding of leishmaniasis and the wound response is less than complete and the influence of vector-derived products is largely unknown.

Through this study we have gained a systems-wide appreciation of the innate immune responses induced in the skin of mice after intradermal injection of PSG. We find that PSG can modulate early innate pathways involved in response to a wound and show that PSG can potently accelerate wound healing in skin. This striking new feature of PSG was controlled by insulin growth factor 1 (IGF1). Using antibody blockade, IGF1 was shown to influence both the wound-accelerating properties of PSG and its disease-exacerbating properties during *Leishmania* infection by sand fly bite.

## Materials and methods

### Ethics statement

All procedures involving animals were approved by the ethical review committees of the London School of Hygiene and Tropical Medicine (PPL: 70/8427), Imperial College (PPL: 70/6712) and the Liverpool School of Tropical Medicine (PPL: 40/2958) and performed in accordance with United Kingdom Government (Home Office) and EC regulations. Mice were anaesthetized by intraperitoneal injection of ketamine 150 mg/kg and xylazine 15 mg/kg, and all efforts were made to minimize any suffering.

### Parasite preparation and inoculation into skin

*Leishmania (Leishmania) mexicana* (MNYC/BZ/62/M379) promastigotes were grown until exponential phase in M199 culture medium (HEPES modification with Earle’s salts, Sigma-Aldrich) supplemented with 10% v/v heat-inactivated foetal calf serum (hiFCS, Gibco, UK), 1% v/v penicillin-streptomycin (PS), 1 x BME vitamins (Sigma-Aldrich), pH 7.2 and then passaged at 5 x 10^5^ promastigotes/ml into Grace's insect culture medium (Invitrogen, supplemented as for M199 medium) pH 5.5 to reach stationary phase at 26°C. This method produces a high yield of *L*. *mexicana* metacyclic promastigotes (typically >85%). Metacyclic promastigotes were isolated from stationary phase cultures using a discontinuous density gradient [31]. One thousand metacyclic promastigotes in Dulbecco’s phosphate buffered saline (PBS) were injected intradermally in the ears of 6–8-week old female BALB/c mice, with or without 0.03 μg *L*. *mexicana* PSG. To minimise tissue damage for Affymetrix analysis, parasites and PSG were delivered by nanoinjection with bevelled fine tipped borosilicate glass microneedles (Drummond) attached to a Nanoject II Auto-Nanolitre injector [32] in a total volume of 160 nl, and for all other experiments in 10 μl using a 31 gauge, 0.3 ml insulin syringe and needle. After 4, 6, 24 or 48 hours post-inoculation, mice were humanely euthanized, and ears were recovered using 2 mm diameter punch biopsy, centred on the site of injection, for RNA extraction. In other experiments, the inoculation of ears with 0.5 μg PSG or PBS was followed 6 hours later by a full-thickness, 2 mm diameter punch biopsy to the site of injection. The diameter of the wounds were measured daily and expressed as proportion of the initial wound.

### PSG preparation

Colony reared female *Lutzomyia longipalpis* (Jacobina strain) were infected with *L*. *mexicana* amastigotes freshly isolated from BALB/c rump lesions as previously described [9] and offered to sand flies through a membrane at 2 x 10^6^ amastigotes/ ml in fresh defibrinated rabbit blood (TCS Biosciences—without antibiotics). Flies were maintained at >70% relative humidity and offered 20% v/v sucrose solution to feed ad libitum. PSG was dissected from 7–8 day old fly infections, processed to remove parasites and contaminating proteins by passing through a 0.22 μm pore diameter syringe filter, then heat-treating for 1 hour at 60°C, followed by four freeze-thaws. To ensure minimal batch-to-batch variation, PSG from 200 flies were collected at a time in 400 μl PBS and adjusted to 0.2 μg/μl total protein using the BCA (bicinchoninic acid) protein assay (Pierce). PSG was stored at -20°C until use [12].

### Saliva preparation

Sand fly saliva was obtained from 5 day-old female uninfected flies using the gland-piercing method into PBS [6] and stored at -20°C until use.

### Sand fly bite transmission

Five-day-old *Lutzomyia longipalpis* (Jacobina strain) female sand flies were infected with *L*. *mexicana* amastigotes through by artificial membrane feeding. Blood-fed flies were separated and maintained under a 12-h light:dark cycle at 28–30°C, 80%–95% relative humidity, and supplied with 20% (w/v) sucrose. Anaesthetised, 6–8-week old female BALB/c mice were infected by allowing single infected sand flies to bite the ear.

### RNA extraction

At different time points post-inoculation recovered mouse ears were fragmented using a MiniBeadBeater (1 min at 5000 rpm/min) in 1 ml of lysis buffer with Precellys ceramic 2.8 mm beads, as previously described [33]. RNA isolation was performed with the RNeasy Plus Mini kit (Qiagen, Courtaboeuf, France), according to the manufacturer’s instructions. Evaluation of RNA quality was performed by optical density measurement using a Nanodrop (ThermoFisher Scientific) and/or their integrity was assessed using an Agilent-2100 Bioanalyzer (RNA integrity >9) [34]. RNA were either used for the GeneChip hybridization or were reverse transcribed in cDNA for real-time quantitative PCR (RTqPCR).

### GeneChip hybridization and data analysis

RNAs were hybridized onto Affymetrix Mouse 430_2 GeneChips, using the Affymetrix Two-Cycle Eukaryotic Target Labelling procedure: http://www.affymetrix.com/support/downloads/manuals/expression_analysis_technical_manual.pdf. MIAME-compliant data are available through ArrayExpress and GEO databases (http://www.ncbi.nlm.nih.gov/geo/query/acc.cgi?acc=GSE52101). Gene-level expression values were derived from the CEL file probe-level hybridization intensities using the model-based Robust Multichip Average algorithm (RMA) [35]. RMA performs normalization, background correction and data summarization. Pearson’s correlation coefficient were calculated and a heatmap representation was performed using the Expression Console Software. Local pooled error (LPE) tests [36] were performed to identify significant differences in gene expression between different conditions. The estimated false discovery rate of this analyse was calculated using the Benjamini and Hochberg approach [37] to correct for multiple comparisons. Ingenuity Pathway Analysis (IPA) software v 9.0 (http://www.ingenuity.com) was used identify canonical pathways regulated by PSG presence at 6 hours post-inoculation. Gene Ontology (GO) analysis was also performed to find gene pathways and networks enriched by PSG in the Affymetrix data set at 6 hours post-infection (http://www.geneontology.org). This service connects to the analysis tool from the PANTHER Classification System (http://pantherdb.org), which is maintained up to date with GO annotations.

### Oligonucleotide primers and transcriptional analyses by RTqPCR

Primers were designed using OligoPerfect Designer (Invitrogen Corp. Carlsbad, CA). The primer sequences and melting temperature (Tm) are summarized in S2 Table. RNAs were reverse transcribed in cDNA using random hexamers (Roche Diagnostics) and Moloney Murine Leukaemia Virus Reverse Transcriptase (Invitrogen, Life Technologies). A SYBR Green-based real-time PCR assay (QuantiTech SYBR Green Kit, Qiagen) for relative quantification of mouse target genes [33,38] was performed on a white MicroAmp Optical 384-well 7900HT Fast Real-Time PCR System (Applied Biosystems). Cycle threshold values (Ct) were determined by the Applied Biosystems SDS2.4 Software. Ct values were input into Biogazelle qBasePLUS software, a flexible and open source program for qPCR data management and analysis [39]. For normalisation calculations, candidate control genes were tested [40] with geNorm [41] and Normfinder programs [42]. *Nono* and *l19* were selected as the most stable reference genes for the BALB/c ears and *ywhaz* and *nono* for BALB/c bone-marrow macrophages.

### Scratch wound model of wound healing

In order to study cell migration in response to PSG-induced inflammation, a simple and robust scratch wound model was used, based on published methods [43]. Mouse derived L929 fibroblast (ATCC) and Kera-308 keratinocyte cell lines (CLS) were cultured in 25 cm^2^ flasks (Costar) in DMEM (Gibco) supplemented with 10% v/v hiFCS, 1% v/v PS, 1 x BME, 2 mM L-glutamine in a 5% CO_2_ humidified incubator at 37°C, and passaged every 7 days. Fibroblasts and keratinocytes were seeded in 24 well plates at a density of 1.5 x 10^5^/ml and 5 x 10^5^/ml per well, respectively, and incubated at 37°C, in a humidified atmosphere of 5% CO for 24 hours to obtain confluent monolayers. Wells were washed gently three times in PBS warmed to room temperature and scraped down the centre using a sterilised 200 μl pipette tip. Following a scratch, wells were washed gently three times in DMEM, and incubated with 100 μl of complete 1% v/v/ hiFCS DMEM-media supplemented with or without 0.5 μg/ml PSG. Positive controls were treated with 10 μg/ml TGFβ2 and 10 μg/ml EGF (Peprotech) [43], and triplicate wells were tested. For orientation, marks were made on the underside of each well along the length of the scratch using permanent marker. A series of photographs were taken in exactly the same position at 0, 12 and 24 hours post-scratch. Photographs were taken using an OptixCam Summit OCS-10.0X 10MP camera on a Nikon inverted microscope, viewed in OCView software (v1.1) and the area of the scratch wound of each image measured in ImageJ using the polygon selection tool (http://rsb.info.nih.gov). This tool was used to outline the leading edge of the monolayer in order to obtain the area of the scratch. The rate of wound closure is expressed as a percentage of the area at time-point zero.

### BrdU incorporation assay

Bromodeoxyuridine (BrdU) is a thymidine analogue that is incorporated into newly synthesized DNA strands of actively proliferating cells. BrdU incorporation was detected immunochemically using the Calbiochem BrdU Cell Proliferation Assay (HTS01), according to the manufacturer’s instructions. Briefly, 20 μl BrdU label was added to each well immediately following the scratch and addition of PSG. At the times indicated post-scratch, wells were incubated with 200 μl BrdU fixative/denaturing solution for 30 min, at room temperature, followed by incubation with 100 μl anti-BrdU antibody (1:100), at room temperature for 1 hour. Wells were washed three times with plate wash solution and 100 μl BrdU peroxidise-conjugated goat ant-mouse IgG (1:1000) added to each well. After 30 min of incubation at room temperature wells were washed three times in plate wash solution and once in dH_2_O before adding 100 μl of fluorogenic substrate solution. Following 30 min incubation in the dark at room temperature, 100 μl stop solution was added and fluorescence intensity was read on a Spectramax M3 plate reader at 320 nm excitation, 460 nm emission. Wells for each treatment were assayed in quadruplicate.

### HeLa/STAT3 luciferase reporter assay

HeLa/STAT3 cells stably express firefly luciferase reporter gene under the control of the STAT3 response element (Signosis). Cells were grown to confluence on square petri dishes in DMEM (high glucose+sodium pyruvate+L-glutamine) (Gibco) supplemented with 1% v/v PS, 10% v/v/ h.i.FBS, 100 μg/ml Hygromycin B in a humidified incubator at 37°C with 5% CO_2_ for 5–6 days. The day before the assay the cells were trypsinised and plated in 96 well plates at 5 x 10^4^ cells per well and incubated as above. Promastigote secretory gel, recombinant human Oncostatin M (Sigma) and anti-IGF1R antibodies (Peprotech) were added to the wells as required and incubated for a further 24 hours. Following this, wells were aspirated and gently washed in PBS once before adding 50 μl of the lysis buffer. Cells were allowed to lyse for 5 mins at room temperature with gentle rocking. Lysing cells were passed up and down a pipette tip before being freeze-thawed once at -80°C and room temperature. Twenty microliters of the lysate was transferred to a new 96 well plate containing 100 μl of a Luciferase substrate (Promega) and mixed gently 2–3 times by pipette. Relative light intensity was measured in a Spectramax M3 plate reader.

### Infection of dermal cells in dermal air pouches

3 ml of sterile air was injected into the backs of shaved BALB/c mice to inflate an air-pouch. Into each air-pouch 1 x 10^6^ *L*. *mexicana* metacyclic promastigotes with or without 1 μg PSG, 1:50 IGF1 Ab (Peprotech), or 1:50 IgG1 isotype control Ab were injected in a total of 150 μl endotoxin-free PBS using a 27-gauge needle. At 48 hours post-injection the cells from the air-pouch were recovered using a 5 ml ice-cold medium cavity lavage. Following this, the cells were concentrated to 0.5 ml by centrifugation (1800 rpm, 5 min) and live cells counted by diluting in trypan blue dye using a Neubauer improved haemocytometer. Following extensive washing to remove extracellular parasites macrophages were separated from neutrophils by adhesion to plastic for 2 hours at 37°C in DMEM medium supplemented with 20% hiFCS. Viable amastigote burdens were determined by transformation assay of amastigotes liberated from 2.5 x 10^5^ cells using the protocol below.

### Bone marrow macrophage culture and infection

Macrophages were differentiated from bone marrow precursor cells in the presence of L929 fibroblast cell culture supernatant as a source of macrophage colony stimulating factor (CSF-1-responsive bone marrow-derived macrophages) as previously described [12]. Briefly, bone marrow was obtained from femurs from 6–8 week old BALB/c mice and bone marrow cells were cultured in supplemented DMEM medium (Gibco) (10% v/v hiFCS, 5% v/v horse serum, 1% v/v PS, 2 mM L-glutamine, 5 x 10^−5^ M β-mercaptoethanol and 15% v/v L929 culture supernatant) at 37°C, 10% CO_2_ in a humidified incubator. Following 8 days of differentiation in plastic petridishes the bone marrow derived macrophages (BMMΦ) were washed and seeded into 16 chambered slides at 5 x 10^4^ BMMΦ/200 μl (Lab-Tek, Nunc) for promastigote transformation assays, or in 24 well culture plates at 5 x 10^5^/ml for arginase assays and RTqPCR. *In vitro* generated macrophages were exposed to *Leishmania* at a parasite to macrophage ratio (multiplicity of infection) of 5 *L*. *mexicana* metacyclic promastigotes to 1 macrophage (MOI 5:1) for 4 hours to achieve an intermediary level infection to assess the impact of various activations or vector-derived components on infection. Macrophages were washed extensively in DMEM to remove external parasites, then activators (20 U/ml IL-4, Preprotech) or *L*. *mexicana* PSG (0.5 μg for 5 x 10^5^ MΦ and 0.25 μg for 5 x 10^4^ MΦ) were added and the cells were cultured for up to a further 48 hours. The average viable intracellular parasite load was determined by promastigote transformation growth assay, below. RNA were extracted from macrophages using the RNeasy Plus Mini Kit (Qiagen) and the samples were reverse transcribed as previously described [44].

### Promastigote transformation growth assay

5 x 10^5^ BALB/c BMMΦ or 2.5 x 10^5^ dermal macrophages recruited to and infected for 48 hours in air-pouches were lysed to release their amastigotes and grown for a further 48 hours in promastigote medium. Macrophages were lysed with sterile 0.008% v/v SDS in simple DMEM medium for 5 minutes at room temperature. The lysis was stopped by the addition of 17% v/v hiFCS in simple DMEM medium and the cells were mechanically ruptured with a cell scraper. Released amastigotes were concentrated on the bottom of the wells by centrifuging the plate at 3100 rpm for 10 minutes, washed in PBS and resuspended in 200 μl M199 promastigote medium (M199 medium supplemented with 1% v/v PS, 20% v/v hiFBS, 1% v/v hemin, 1 x BME vitamins, 4.2 mM NaHCO3, pH 7.2). The density of viable amastigotes that transformed and grew as promastigotes after 96 hours of culture at 26°C in 96 well microtitre plates was counted by haemocytometer.

### Arginase assay

Arginase activity was determined by measuring the amount of urea generated from the hydrolysis of L-arginine as described [23]. Briefly, 5 x 10^5^ BMMΦ were solubilised with 200 μl lysis solution (0.1% Triton X-100 with protease inhibitor cocktail tablets (Roche), 10 mM MnCl_2_, 25 mM Tris-HCl) and the enzyme was activated by heating for 10 min at 56°C. To this 10 μl 0.5 M L-arginine (pH 9.7) was added to the preparations to allow hydrolysis of the substrate and incubated at 37°C for 15–120 min. The reaction was stopped with 400 μl of H_2_SO_4_, H_3_PO_4_, H_2_O (1:3:7 v/v). The urea concentration was measured at 550 nm after the addition of 20 ml α-isonitrosopropiophenone (dissolved in 100% ethanol), followed by heating at 100°C for 45 min. One unit of enzyme activity is defined as the amount of enzyme that catalyses the formation of 1 μmol of urea per min.

### Luminex

Skin biopsies were homogenized in 100 μl of lysis solution (Fisher Scientific) containing T-PER Tissue Protein Extraction Reagent, 1.0 mM dithiothreitol and 1:100 protease inhibitor cocktail III using a tissue homogenizer (Biomasher, Camlab) and diluted with Bio-Plex samples diluents. IL-13 and IL-17a levels were determined using a Bio-Plex Mouse cytokine assay (Biorad) according to the manufacturer’s protocols. Briefly, antibody-coupled beads (50 μl) and the tissue homogenate (50 μl) were added into each well of 96-well filter plate. After incubation at room temperature for 30 minutes with agitation on a plate shaker, the supernatants were washed three times by wash buffer. The detection antibodies (25 μl) were added, and incubated at room temperature for another 30 minutes on plate shaker. Finally, streptavidin—PE (50 μl) was added into each well and incubated at room temperature for 10 minutes before washing and then 125 μl of Assay Buffer was added into each well. The microspheres were quantified using a Luminex 100 System (Luminexcorp).

### Statistical analyses

Mann Whitney t-test was used to test the statistical significance between groups using GraphPad PRISM (v 5.0). All comparisons were made to the saline control except those indicated on the graphs with a connecting line. Linear correlation coefficients were generated by Spearman-rank correlation to assess the association of sand fly transmitted dose with host cytokine expression at the bite site. Significance was considered as p<0.05.

## Results

### PSG induces rapid expression of many genes in *L*. *mexicana* infected BALB/c mouse skin associated with different innate immune pathways and wound healing processes

**Fig 1:**
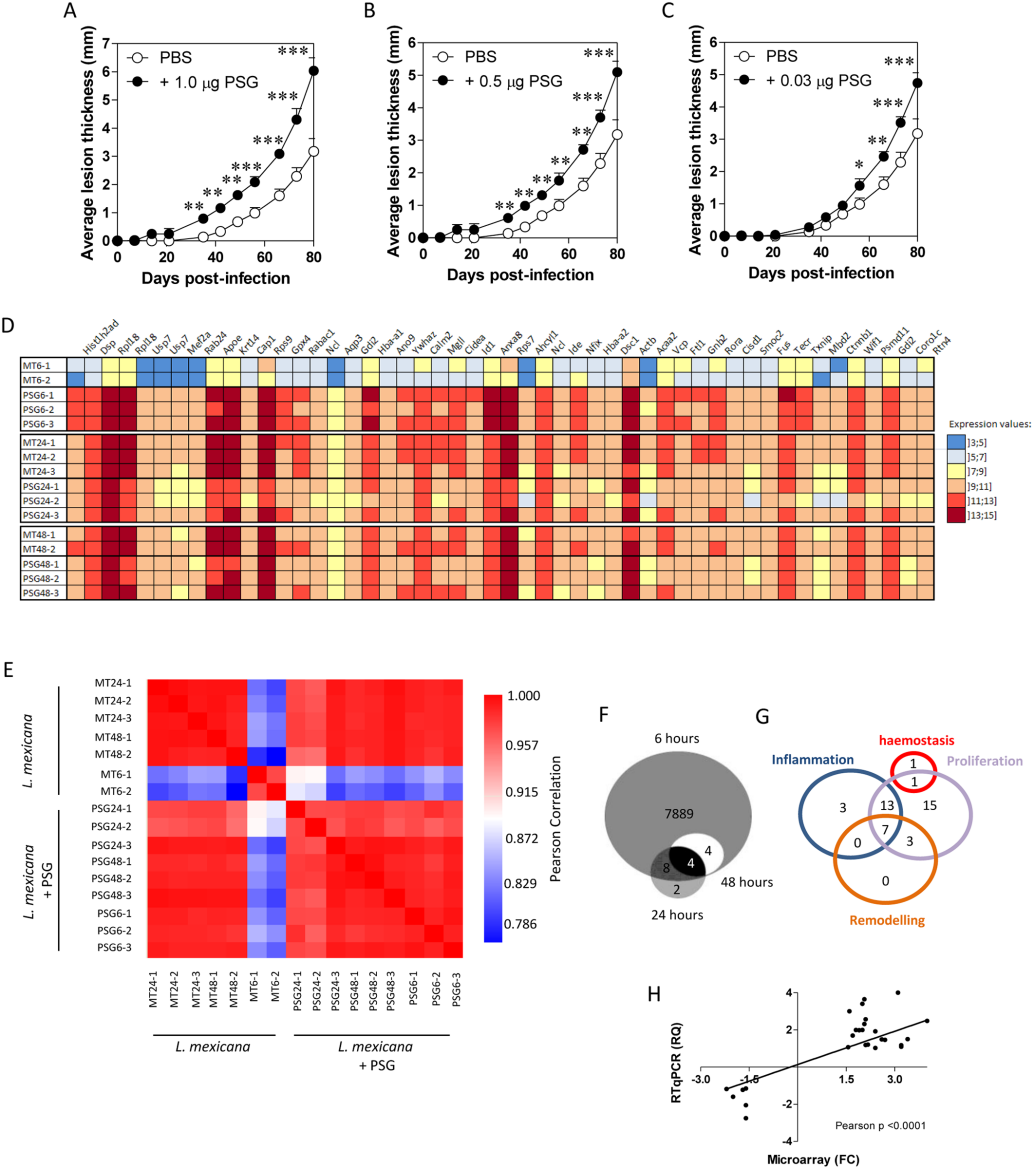
PSG exacerbates *Leishmania* in skin over a wide physiological range and induces different gene modulation at different time points post-infection in skin. (A-C) BALB/c mice were infected with 1 x 10^3^ *L*. *mexicana* metacyclic promastigotes with or without 1.0 μg (A), 0.5 μg (B) or 0.03 (C) μg *L*. *mexicana* PSG, s.c in to the left footpad and the evolution of the lesions, compared to the contralateral foot, recorded over 80 days. Final average parasite burdens ± S.D. for these lesions: PBS: 5.167 x 10^7^ ± 9.79 x 10^6^; 1.0 μg PSG: 4.74 x 10^8^ ± 3.21 x 10^8^; 0.5 μg PSG: 3.27 x 10^8^ ± 3.59 x 10^8^; 0.03 μg PSG: 1.96 x 10^8^ ± 3.05 x 10^8^ amastigotes per footpad. BALB/c mice received co-inoculation of either PBS or 0.03 μg PSG with 1 x 10^3^ *L*. *mexicana* metacyclic promastigotes between the dermal sheets of the ear pinna using a nanoinjector. D) Heat map representation of normalised expression values. Genes shown are the top 50 differentially expressed at 6 hours (FC >1.5 at 5% FDR). E) Heat map representation of the Pearson correlation coefficients between replicates for all modulated genes. The colour gradient extends from red, representing perfect correlation (correlation distance, r, of 1), to cyan for low correlation. Chip coding: Experiment/Time/Replicate number, e.g. MT6-2 represents the 2^nd^ chip from a control ear injected with PBS and parasites, sampled at 6 hours post-infection; chips coded PSG are from PSG-inoculated ears. (F and G) Venn diagrams of the number of overlapping genes differentially expressed (FC > 1.5 at 5% FDR) at 6, 24 and 48 hours between ears injected with PBS and metacyclic promastigotes versus PSG and metacyclic promastigotes. F) Total number of modulated genes. G) Genes involved in different phases of wound healing. H) Validation of microarray gene expression following i.d. injection of 0.5 μg PSG per ear. Real-time quantitative PCR of 34 randomly selected up- and down-regulated genes, among the differentially expressed gene list of 6 hours p.i. (FC>1.5, FDR 5%).

Using a low dose model of infection with physiologically relevant concentrations of PSG, we compared the transcriptional profiles of BALB/c ears 6, 24 and 48 hours post-inoculation (p.i.) of 1 x 10^3^ *Leishmania (L*.*) mexicana* metacyclic promastigotes with and without 0.03 μg of PSG. Prior to this, footpad infections confirmed that PSG was able to exacerbate *L*. *mexicana* over a wide range of doses, including 0.03 μg, found to be egested by infected sand flies [12] (Fig 1A–1C). Sand flies are pool-feeding vectors, which lacerate the upper dermal capillary bed with their barbed mouthparts to obtain blood. As a result, the damage made to the skin is disproportionately larger than other vessel-feeding vectors with hypodermic-like mouthparts (e.g. mosquitoes), yet the damage is still very focal. This is hard to replicate using needles since even the smallest (34 gauge) are some 9.5 times bigger than a sand fly’s piercing-proboscis [45], which will lead to extensive tissue damage. Furthermore, studies that use the intradermal route of needle-infection to the ear typically inject 10–20 μl, which separates the two dermal sheets over a large area of the ear, causing even more damage. Therefore, for our Affymetrix gene transcription study we used nanoinjection to minimise the wound created and volume injected (160 nl), to focus on those interactions driven by the PSG; combined with a dose of PSG (0.03 μg) equivalent to the upper-limit egested by *L*. *mexicana*-infected *Lu*. *longipalpis* [12]. The expression profiles of individual mice after PSG injection were comparable, both in quantity and quality, as illustrated by a heat map of the top 50 responsive genes (Fig 1A) and from the high degree of correlation in expression levels between mice after PSG administration (Fig 1B).

Out of a total of 28,853 mouse genes assayed 7,907 with known function showed ≥1.5-fold change (FC) at a 5% false discovery rate (FDR) that were differentially expressed in response to PSG. The vast majority of these genes (99.8%) were expressed during the first 6 hours of infection (Fig 1C). Focussing on this early time point, we subjected the transcripts to a biological interaction network analysis using the Ingenuity Pathway Analysis (IPA) software v 9.0. Using a cut-off of ≥FC5 at 5% FDR, 5,312 genes with known function were used to analyse the most significant canonical pathways.

The top 168 most enriched pathways are listed in Supplementary Table 1 (S1 Table), where a greater—ln (p-value) reflects increasing enrichment of PSG targets within a pathway. Of these 43 (26%) (7.94E-16 < p value < 5.00E-2; FC5, 5% FDR) are involved in innate immunity and one or more phases of wound healing (Table 1). Among these, 2 are involved in haemostasis, 24 in inflammation, 36 in epithelial cell proliferation and 10 in fibrosis and wound remodelling (Fig 1D). Gene Ontology (GO) analysis of the Affymetrix data revealed many genes involved in growth factor signalling and polyamine metabolism were enriched in skin by the presence of PSG (S2 Table). Thus, the majority of genes modulated by PSG following infection with *L*. *mexicana* are involved in the inflammation and proliferation phases of wound healing. [46]. The proliferation phase can be further sub-divided into: (i) angiogenesis, (ii) fibroplasia and granulation tissue formation, (iii) epithelialisation and (iv) contraction. Among the 39 pathways involved in the proliferation phase, 14, 27, 29 and 5 differentially expressed genes are involved in these four sub-phases, respectively. Thus, at 6 hours p.i. PSG induces different modulated canonical pathways predominantly involved in: (i) inflammation, (ii) fibroplasias and granulation tissue formation and (iii) epithelialisation.

**Table 1:** In skin, PSG induces gene pathways involved in wound healing.

canonical pathways	P Value	Percentage	phase of wound healing
**mTOR**	7.94E-16	19.4	angiogenesis; fibroplasias and granulation; epithelialisation
**hypoxia signalling in the cardiovascular system**	5.25E-09	25.0	angiogenesis; fibroplasias and granulation; epithelialisation
**IGF-1 Signalling**	2.51E-05	15.0	fibroplasias and granulation; epithelialisation
**Production of NO and ROS in macrophages**	2.63E-05	11.0	inflammation; angiogenesis; fibroplasias and granulation; epithelialisation; remodelling
**RhoA signalling**	3.63E-05	14.9	inflammation; fibroplasias and granulation; epithelialisation
**ERK5 signalling**	4.68E-05	18.8	epithelialisation
**actin cytoskeleton**	5.62E-05	10.5	epithelialisation; contraction
**NRF2-mediated oxidative stress response**	7.76E-05	11.5	inflammation
**ILK signalling**	7.76E-05	11.4	fibroplasias and granulation; epithelialisation
**integrin signalling**	3.16E-04	10.5	fibroplasias and granulation; epithelialisation; contraction; remodelling
**p53 signalling**	5.89E-04	13.5	inflammation; fibroplasias and granulation; remodelling
**glucocorticoid receptor signalling**	1.17E-03	8.5	epithelialisation
**calcium signalling**	1.91E-03	8.7	haemostasis; inflammation; fibroplasias and granulation; epithelialisation; contraction
**Cdc42 signalling**	2.00E-03	8.3	epithelialisation
**NGF signalling**	2.19E-03	11.2	inflammation; fibroplasias and granulation; epithelialisation
**thrombin signalling**	3.39E-03	9.2	haemostasis
**androgen signalling**	3.63E-03	9.0	inflammation; epithelialisation
**HGF signalling**	4.07E-03	11.4	epithelialisation
**FAK signalling**	4.79E-03	10.8	epithelialisation
**apoptosis signalling**	5.25E-03	11.5	inflammation; fibroplasias and granulation; remodelling
**arginine and proline metabolism**	8.13E-03	5.1	inflammation; fibroplasias and granulation; epithelialisation; remodelling
**EGF signalling**	8.51E-03	13.5	epithelialisation
**IL-6 signalling**	8.71E-03	11.0	inflammation; fibroplasias and granulation; epithelialisation
**Gap junction signalling**	1.05E-02	8.2	epithelialisation
**FGF signalling**	1.05E-02	11.1	angiogenesis; fibroplasias and granulation; epithelialisation; contraction; remodelling
**VEGF signalling**	1.05E-02	10.1	angiogenesis; fibroplasias and granulation; epithelialisation
**PDGF signalling**	1.07E-02	11.4	inflammation; angiogenesis; fibroplasias and granulation; epithelialisation; contraction; remodelling
**Wnt/β-catenin signalling**	1.20E-02	9.2	fibroplasias and granulation; contraction
**CCR5 signalling in macrophages**	1.29E-02	8.5	inflammation; epithelialisation
**IL-4 signalling**	1.70E-02	11.0	fibroplasias and granulation; remodelling
**IL-1 signalling**	1.70E-02	9.4	inflammation
**IL-2 signalling**	1.82E-02	12.1	inflammation; angiogenesis; fibroplasias and granulation; epithelialisation
**regulation of IL-2 in T-lymphocytes**	1.91E-02	10.1	inflammation; angiogenesis; fibroplasias and granulation; epithelialisation
**PPAR signalling**	1.95E-02	9.4	inflammation; fibroplasias and granulation; epithelialisation; remodelling
**IL-10 signalling**	2.19E-02	10.3	inflammation; fibroplasias and granulation
**acute phase response signalling**	2.24E-02	8.4	inflammation
**IL-8 signalling**	2.24E-02	7.8	inflammation; epithelialisation
**IL-17 signalling**	2.34E-02	10.8	inflammation
**chemokine signalling**	2.34E-02	11.0	inflammation; angiogenesis; fibroplasias and granulation; epithelialisation
**IL-15 signalling**	3.72E-02	10.4	inflammation
**GM-CSF signalling**	3.72E-02	10.4	inflammation; angiogenesis; fibroplasias and granulation; epithelialisation
**PTEN signalling**	4.68E-02	8.1	inflammation; fibroplasias and granulation; remodelling
**angiopoietin signalling**	5.00E-02	9.5	angiogenesis

Top canonical pathways involved in innate immunity and one or more phases of wound healing (7.94E-16 < p value < 5.00E-2; FC5, 5% FDR) from BALB/c ear dermis microinjected with 0.5 μg *L*. *mexicana* PSG expressed at 6 hours post-inoculation.

### Validation of gene expression profiling

To validate the microarray data, we selected 35 genes associated with wound healing with different abundance levels (S3 Table) and quantified them by Real-Time reverse transcription quantitative PCR (RTqPCR) from samples collected at 6 hours p.i. All primers had amplification efficiencies close to 2. All threshold cycle (CT) values were normalized with two reference genes, *nono* and *l19*, that tested as the most stable. RTqPCR confirmed a high correlation (p<0.0001) with the corresponding microarray expression. (Fig 1H).

### PSG accelerates wound healing in skin

**Fig 2:**
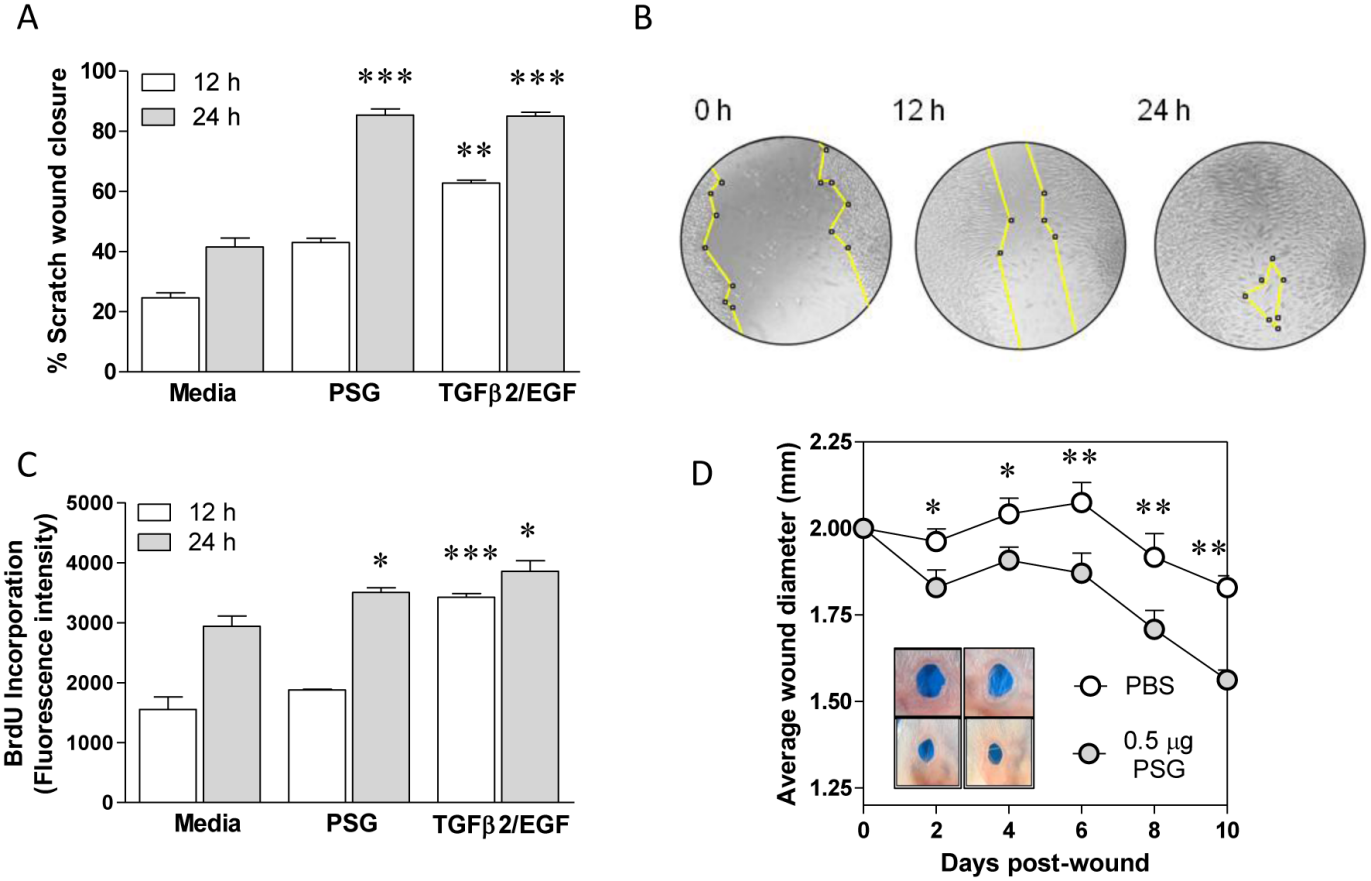
PSG improves wound healing *in vitro* and in skin. Monolayers of L929 fibroblasts were scratched in the presence of culture media supplemented with or without 0.5 μg/ml *L*. *mexicana* PSG. Positive controls were treated with 10 μg/ml TGFβ2 and 10 μg/ml EGF. A) At 0, 12 and 24 hours post-wound, photomicrographs were taken and scratch closure was determined from using ImageJ. B) Representative images for the closure of a PSG-treated scratch. C) In replicate experiments, cell proliferation of fibroblasts was determined by measuring the incorporation of the fluorescent thymidine analogue, BrdU. Statistical analyses were performed between Media vs. PSG at each time point. Each in condition was performed in quadruplicate, data is pooled from 3 experiments. D) Daily wound closure following intradermal injection of PBS (open symbols) or 0.5 μg *L*. *mexicana* PSG (closed symbols) in the ears of BALB/c mice. Ears were wounded with a 2 mm diameter full-thickness ear punch 6 hours after the intra-dermal injection of PBS or PSG. Inset photographs are ears representative of each group at day 10 post-wound. Average wound closure ±SEM is shown from 12 mice per group (*: p<0.05; **: p<0.005; ***: p<0.0005 by Mann Whitney *t*-test); data is pooled from duplicate experiments.

As PSG up-regulates transcripts involved in wounding, when inoculated together with *L*. *mexicana* metacyclics, we hypothesized that PSG could promote wound healing. In a series of *in vitro* and *in vivo* experiments, we tested the ability of PSG to influence some of the key stages in skin injury and repair. We adapted an *in vitro* model of wounding using fibroblast and keratinocyte cell lines monolayers to test the effect of PSG on epithelial cell proliferation and migration [42]. Using a scratch wound model, we measured cell proliferation (BrdU incorporation) and cell migration over a 24 hour period (Fig 2A to 2C). The addition of PSG resulted in accelerated scratch closure (Fig 2A & 2B) following enhanced cell proliferation (Fig 2C) and migration of fibroblasts. In contrast, PSG did not exert any effect on keratinocyte monolayers (S1 Fig). To translate these *in vitro* results to wound healing in skin, we conditioned mouse ears with the intradermal injection of 0.5 μg PSG or PBS 4 hours prior to making 2 mm diameter full-thickness wounds and measured wound closure over 10 days (Fig 2D). PSG accelerated wound healing from day 2 onwards, leading day on day to significantly smaller wounds when compared to the PBS-injected controls (p<0.05).

### PSG augments the early inflammatory response of the dermis to injury

**Fig 3:**
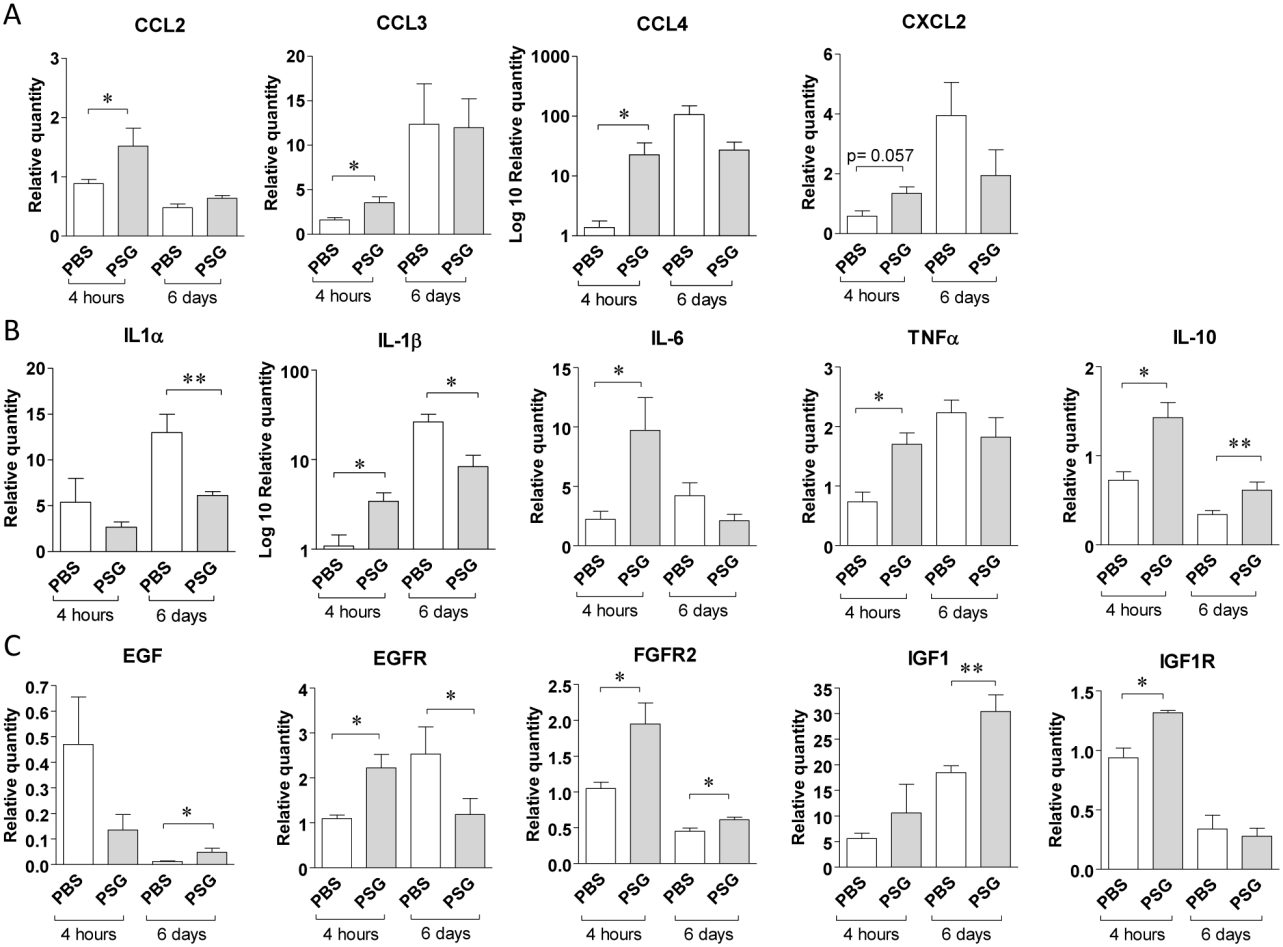
The presence of PSG in a dermal wound differentially modulates the expression of genes involved in various stages of healing. Ears of BALB/c mice were intra-dermally inoculated with 0.5 μg *L*. *mexicana* PSG (grey bars) or PBS (white bars) 4 hours prior to a full-thickness 2 mm diameter punch biopsy. A-C) 4 hours and 6 days post-wound ears were measured for transcripts involved in the inflammation and cell proliferation phases of wound healing by real-time quantitative PCR. A) Chemokines: CCL2, CCL3, CCL4 and CXCL2, B) pro-inflammatory-modulating cytokines: IL-1α, IL-1β, IL-6, IL-10 and TNFα, and C) epidermal growth factors and receptors: EGF, IGF1, EGFR, IGF1R and FGFR2. Relative expression was normalised to the housekeeping genes *nono* and *l19* and is presented as the mean ±SD with 9–12 mice per group (*: p<0.05, **: p<0.005 by Mann Whitney *t*-test).

Next, we investigated the role of PSG to modulate chemokines, cytokines and growth factors in skin over the course of a host’s response to a wound. We found that PSG strongly upregulated the early expression and secretion of a wide range of chemokines and cytokines in BALB/c ear dermis following a full-thickness wound (Fig 3A & 3B). Previously, we demonstrated that *L*. *mexicana* PSG rapidly (within 4 hours) recruits both neutrophils and macrophages to BALB/c mouse dermal air-pouches; and this was synergistic with *Lu*. *longipalpis* sand fly saliva [12]. Here we extend this finding by demonstrating that within 4 hours PSG upregulated the expression of the macrophage-recruiting chemokines CCL2 (MCP-1), CCL3 (MIP-1α), CCL4 (MIP-1β), and the neutrophil-recruiting chemokine CXCL2 (KC) in skin following a wound (Fig 3A). Similarly, PSG also increased the expression of a large number of pro-inflammatory cytokines, including IL-1β, IL-6and TNFα (Fig 3B). Collectively, these results show that PSG favours an early pro-inflammatory response in skin.

Macrophages provide a large proportion of pro-inflammatory cytokines [reviewed by 46] and their presence is not only important for the control of inflammatory cell migration but also for the stimulation of fibroblast and keratinocyte proliferation [47]. All the pro-inflammatory cytokines (IL-1α, IL-1β, IL-6, TNFα) fell in the PSG-treated ears 6 days into wound healing. This may result from the PSG’s ability to promote macrophage alternative activation [12]. Alternatively activated (M2) macrophages (AAMΦ) predominate during the resolution phase of wound healing and secrete IL-10, an inhibitor of neutrophil and macrophage infiltration toward the site of injury [48]. During leishmaniasis, IL-10 plays an important role in suppressing the immune response and maintaining parasite persistence [49]. In the ears of BALB/c mice we found an early 7-fold increase in the expression of IL-10 (p = 0.052) in response to PSG following a wound, and by 6 days post-wound IL-10 was the only cytokine that remained upregulated (Fig 3B). IL-10 produced by resident skin cells [48] can inhibit the production of CCL2 and CCL3 chemokines and the pro-inflammatory cytokines IL-1β, IL-6 and TNFα [47,50]. Therefore, IL-10 may act as a negative regulator of the inflammatory response to promote the proliferative phase of wound healing.

### PSG promotes the conditions for proliferation of fibroblasts and epithelial cells in skin

During the epithelialisation phase of wound healing, keratinocytes migrate to the wound edge and proliferate to form a new epidermis [51–55]. Re-epithelialisation is under the tight control from a variety of growth factors. Affymetrix data and IPA analysis revealed that after 6 hours of injection of PSG into the dermis, epidermal growth factor (EGF), vascular endothelial growth factor (VEGF) and hepatocyte growth factor (HGF) signalling pathways were enhanced (Table 1). This was supported by GO analysis, which revealed a wide range of growth factor signalling pathways to be enriched by PSG, including: VEGF, fibroblast growth factor (FGF), platelet derived growth factor (PDGF), Wingless-related integration site (Wnt), insulin growth factor-1 (IGF1), EGF and Nerve growth factor (NGF) (S2 Table). By 4 hours post-wound, TNFα, EGF and EGFR expression increased 4-, 2- and 1.5-fold, respectively, compared to the PBS controls (Fig 3B and 3C). EGF acts in a paracrine fashion on keratinocytes by increasing their proliferation and their migration in wounds [51,52]. The influence of PSG in skin was to show a general increase in the expression of EGF and its receptor EGFR (Fig 3C). VEGF is expressed in keratinocytes [53] and is induced by HGF [54], resulting in increased migration and epithelialisation [55]. Inside a wound, AAMΦ initiate angiogenesis by releasing TNFα and VEGF [53].

In the proliferation phase of wound healing fibroblasts migrate, proliferate and synthesize extracellular matrix components including type I collagen [56]. This phase is induced by different cytokine and growth factors such as transforming growth factor beta (TGFβ), PGDF, FGF and IGF1 [43,56]. These signalling pathways were significantly upregulated in the presence of PSG (Table 1). In addition, some cytokine pathways involved in fibroplasia and granulation tissue formation were upregulated by the presence of PSG (Table 1). In particular, IL-4 and IL-6 signalling favour the synthesis of extracellular matrix molecules, including type I collagen and FGF and IGF1 promote the recruitment and differentiation of fibroblasts, and the production of extracellular matrix components [44,57,58]. Transcripts of FGFR2, IGF1 and IGF1R were significantly upregulated in presence of PSG (Fig 3C). Interestingly, we observed that in wounds PSG enhanced the early expression of the IGF1 receptor, followed six days later by enhanced the expression of IGF1 (Fig 3C); indicating that IGF1-signalling was promoted throughout the whole healing process.

### PSG enhances the arginase activity of macrophages, production of arginase-inducing mediators and *Leishmania* survival

**Fig 4:**
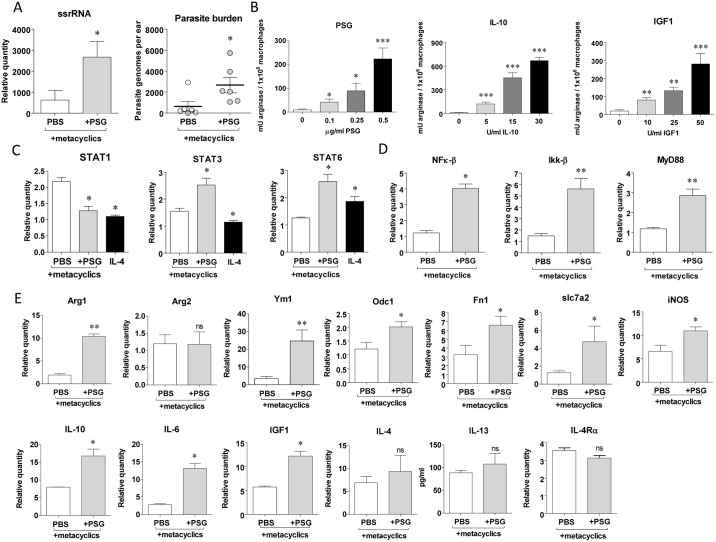
The presence of PSG during an early *Leishmania* infection promotes the expression of genes and signalling pathways involved in macrophage alternative activation. Ears of BALB/c mice were intra-dermally infected with 1 x 10^3^ *L*. *mexicana* metacyclic promastigotes with 0.5 μg *L*. *mexicana* PSG (grey bars) or PBS (white bars), or 5 x 10^5^ BMMΦ from BALB/c mice were exposed to *L*. *mexicana* PSG or PBS with or without *L*. *mexicana* metacyclic promastigotes (MOI 5:1). A) 48h post-infection expression of *Leishmania ssrRNA* in ears and the extrapolated parasite burdens, n = 6/group. B) Arginase activity of uninfected BMMΦ exposed to increasing concentrations of PSG, IL-10 or IGF1 for 48 hours. C) 48 h expression of signal transducer and activator of transcription (STAT) factors 1, 3 and 6 in 5 x 10^5^ infected BMMΦ in response to PBS, 0.5 μg/ml PSG or uninfected BMMΦ exposed to 20 U/ml recombinant murine IL-4. D) 48 h expression of STAT-signalling transcription factors: NF-κB, Ikk-β and MyD88 in infected BALB/c macrophages co-incubated with PBS or 0.5μg PSG.E) 48 h expression of markers of macrophage alternative activation or arginine metabolism measured in infected BALB/c ears in response to intradermal injection of PBS or 0.5 μg/ml PSG by real-time quantitative PCR. IL-13 production was determined from whole cell lysates using Luminex. Data is representative of triplicate experiments. Relative expression was normalised to the housekeeping genes *ywhaz* and *nono* and is presented as the mean ±SD (*: p<0.05, **: p<0.005 by Mann Whitney *t*-test).

**Fig 5:**
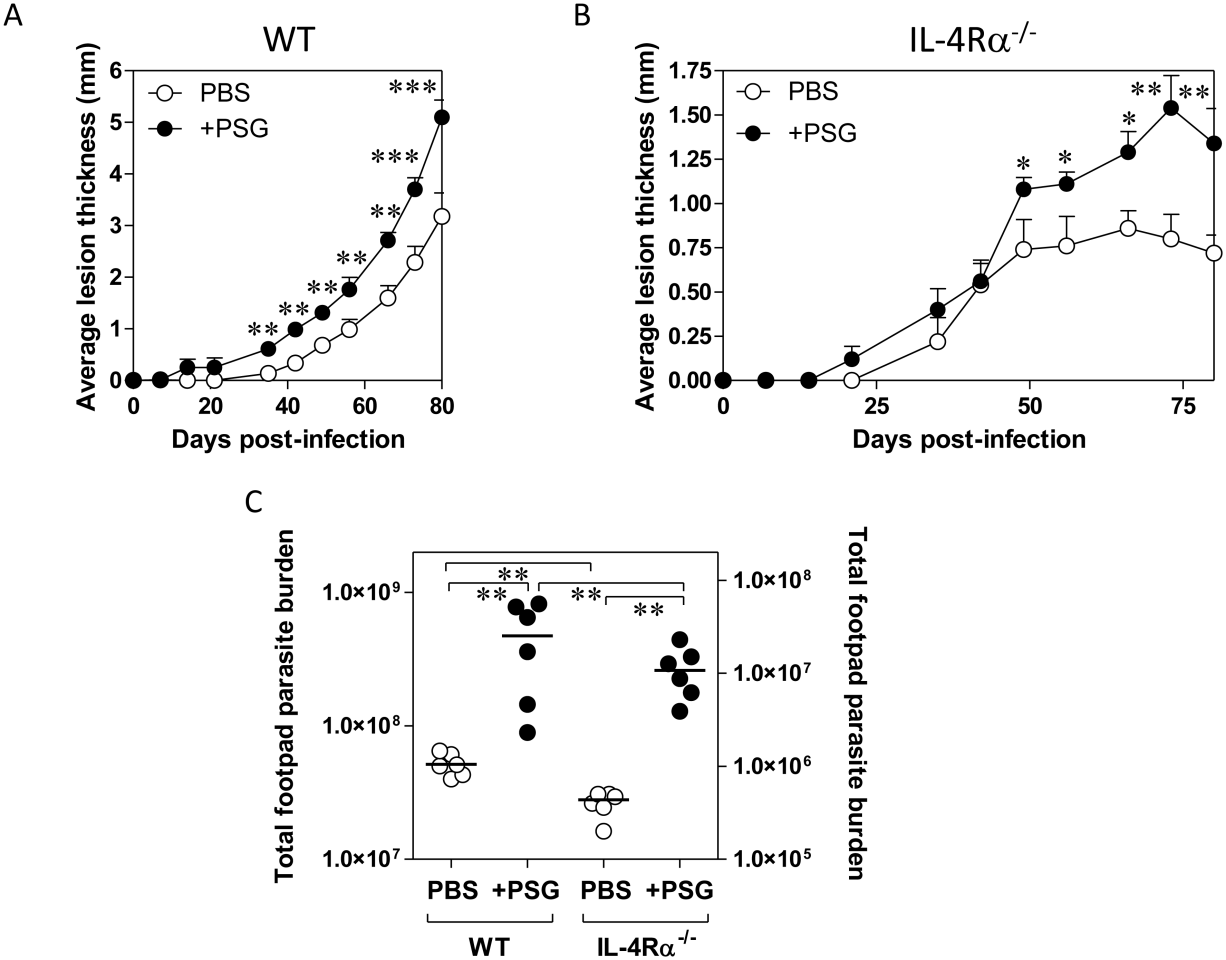
PSG does not require IL-4Rα signalling to exacerbate cutaneous leishmaniasis in mice. A and B) Footpad infections of WT (A) and IL-4Rα^-/-^ (B) BALB/c mice with 1 x 10^3^ *L*. *mexicana* metacyclic promastigotes intradermally injected with PBS or 0.5 μg *L*. *mexicana* PSG, 6 mice per group. C) Final parasite burdens of footpads from B) at the end of the experiment (WT: 80 d post-infection, IL-4Rα^-/-^: 151 d post-infection). Data pooled from two replicate experiments. A&B) Averages ±SEM are shown, C) Each point represent individual mice, bars indicate the mean (*: p<0.05; **: p<0.005; ***: p<0.0005 by Mann Whitney *t*-test).

Alternatively activated macrophages and L-arginine metabolism are instrumental for efficient wound healing and *Leishmania* growth in the vertebrate host [44,57,58]. To assess whether PSG promoted the viability of parasites in the early intradermal injection site we measured small subunit ribosomal RNA (*ssrRNA*) parasite transcripts by RTqPCR in the same samples in which mouse transcripts were quantified. At 6 hours post-infection, we could detect an 8-fold increase of parasite transcripts in those ears co-injected with PSG, translating to a 4.3-fold increase in parasite burden (Fig 4A). From our Affymetrix and RTqPCR results of PSG inoculated into skin we found that PSG significantly increased the early expression of IL-10 and IGF1 (9 and 4-fold change respectively), both known inducers of arginase activity in macrophages [59,60]. This was confirmed *in vitro* when unstimulated bone-marrow macrophages (BMMΦ) were co-incubated with increasing amounts of PSG, IL-10 or IGF1 for 48 hours (Fig 4B). Arginase 1 in murine macrophages can be induced by Th2 cytokines or pathogen-induced TLRs via signal transducer and activator of transcription (STAT) factors STAT6 and STAT3, respectively [61,62]. Expression of classically activated/M1- and alternatively activated/M2-specific STAT transcription factors confirmed that BMMΦ exposed to the highest concentration of PSG suppressed interferon gamma (INF-γ)-responsive STAT1 and enhanced STAT6 and STAT3 (Fig 4C). This was associated with increases in the expression of STAT-signalling transcription factors: nuclear transcription factor kappa-B (NF-κB), inhibitor of nuclear factor kappa-B kinase subunit beta (IKK-β) and the TLR adaptor protein myeloid differentiation primary response gene 88 (MyD88)—used by almost all TLRs to activate NF-κB (Fig 4D). Exposure of STAT3 luciferase reporter HeLa cells to 1 μg PSG for 24 hours resulted in 3.1-fold enhanced luciferase activity, showing that the JAK-STAT receptor signalling pathway was activated by PSG and this could be partially blocked by inclusion of antibodies against the IGF1 receptor (S2 Fig). These data indicate that PSG promotes the alternative activation of macrophages in skin. Supporting this, we could detect an increased expression of many markers of alternatively activated myeloid cells in BALB/c ears 24 hours post-infection with or without PSG, namely: arginase 1 (Arg1) (but not mitochondrial arginase 2, Arg2), ornithine decarboxylase 1 (Odc1), chitinase-like protein 3 (Ym1), fibronectin 1 (Fn1); Arg1-inducers: IL-10, IL-6 and IGF1; L-arginine transporter Slc7a2 and L-arginine metabolism: inducible nitric oxide synthase (iNOS); but not expression of the prototypical alternative activator IL-4, production of IL-13 protein or expression of the IL-4/13 receptor IL-4Rα (Fig 4E). To test the role of IL-4 or IL-13 for the action of PSG during cutaneous leishmaniasis we co-infected WT and IL-4Rα^-/-^ BALB/c mice with a low dose of *L*. *mexicana* metacyclics in the presence or absence of PSG (Fig 5). This showed that PSG could exacerbate cutaneous lesion pathology of *L*. *mexicana* (Fig 5A & 5B) and promote parasite replication (Fig 5C) in the absence of IL-4 or IL-13 signalling, similar to WT mice. Taken together, these *in vivo* and *in vitro* results reinforce the PSG’s ability to manipulate macrophage alternative activation in favour of early *Leishmania* infection and survival potentially through STAT6 and STAT3 pathways, independently of IL-4Rα-signalling.

### PSG enhances both wound healing and *Leishmania* infection by sand fly bite through insulin-like growth factor 1 signalling *in vivo*

**Fig 6:**
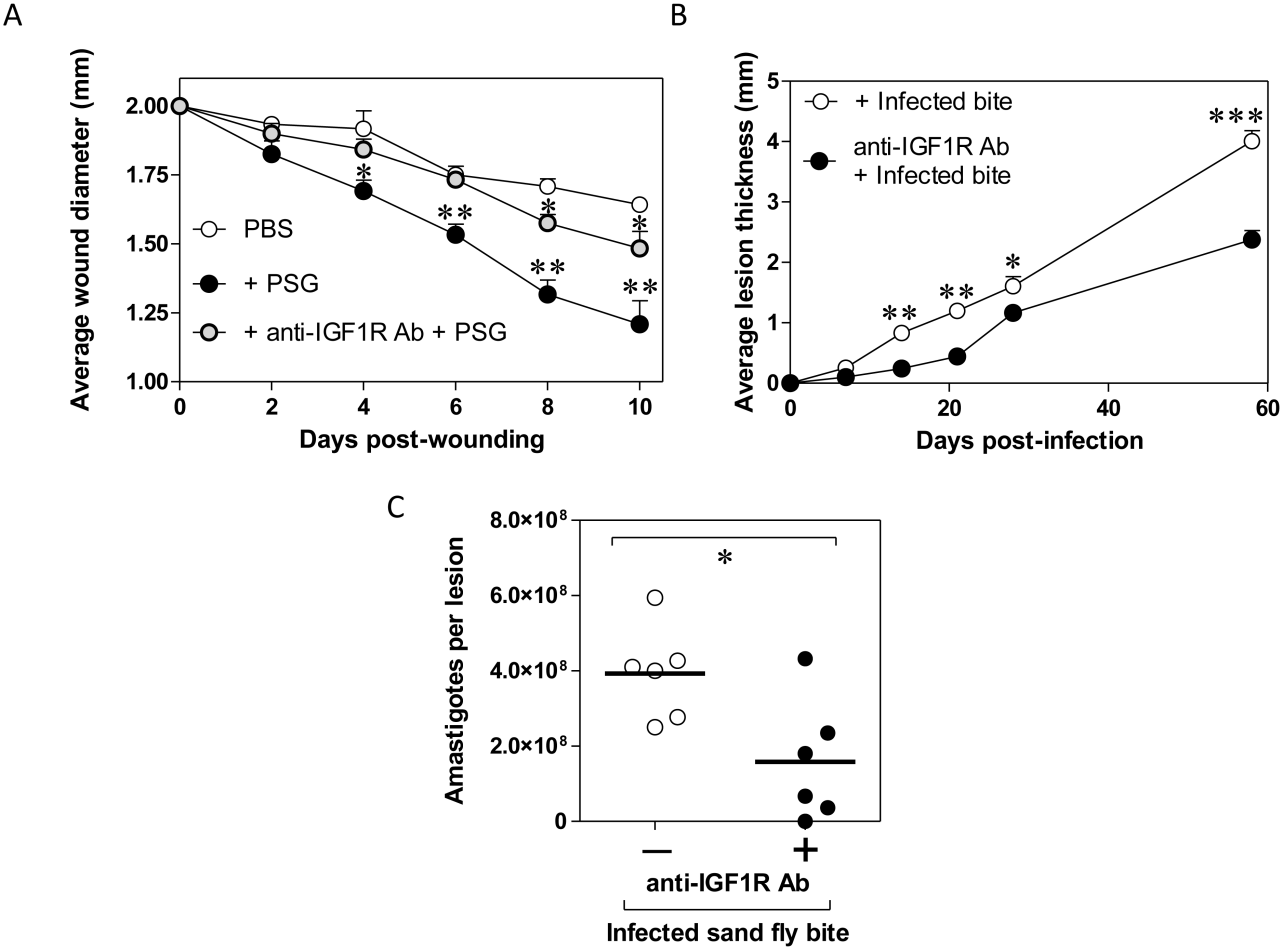
Insulin-like growth factor 1-signalling controls the wound healing and *Leishmania*-exacerbating properties of PSG in mice. A) Rate of 2 mm diameter wound closure in BALB/c ears preconditioned with PBS, 0.5 μg *L*. *mexicana* PSG or 0.5 μg *L*. *mexicana* PSG + 1:50 anti-IGF1R antibody, 12 mice per group. B) Single *L*. *mexicana*-infected sand fly bite infections in footpads of BALB/c mice treated with or without 1:50 anti-IGF1R antibody, 6 mice per group. C) Final parasite burdens of footpads from B) at the end of the experiment. Data pooled from two replicate experiments. A&B) Averages ±SEM are shown, C) Each point represent individual mice, bars indicate the mean (*: p<0.05; **: p<0.005; ***: p<0.0005 by Mann Whitney *t*-test).

Knowing that PSG promoted dermal wound repair and enhanced *L*. *mexicana* infections *in vivo*, we next wanted to test the role of IGF1 on both these outcomes. IGF1 is known to promote the arginase activities of both parasite and host macrophage alike to enhance *Leishmania* infection *in vitro* and *in vivo* [63–65]. From our microarray results, IGF1-signalling was one of the highest-ranking pathways influenced by PSG (Table 1). By combining PSG with anti-IGF1R Ab we could significantly reduce the gel’s ability to promote the healing of 2 mm diameter full-thickness wounds to BALB/c mouse ears (Fig 6A). Furthermore, conditioning skin with anti-IGF1R Ab 4 hours prior to *L*. *mexicana* infection by a single infected *Lu*. *longipalpis* bite (average ± SD, 4.1 x 10^4^ ± 1.18 x 10^4^ promastigotes; 65% ± 17% metacyclics per gut) resulted in a significant delay in cutaneous lesion appearance and consistently weaker lesion growth (Fig 6B). At the end of the experiment, approximately 60 days post-infection, the average lesion size in the antibody-treated mice were some 53% smaller than the isotype antibody controls. Analysis of the parasite burdens of these footpad lesions confirmed that sand fly delivery of *Leishmania* to skin pre-treated with anti-IGF1R Ab significantly reduced their ability to proliferate (Fig 6C, 20-fold decrease, p = 0.021). Collectively, these results show that IGF1-signalling significantly contributes to the PSG’s ability to enhance wound healing and *Leishmania* growth in skin.

### PSG regurgitated during infected sand fly bite promotes IGF1 and IL-10 expression and enhances transmissible macrophage infections via arginase

**Fig 7:**
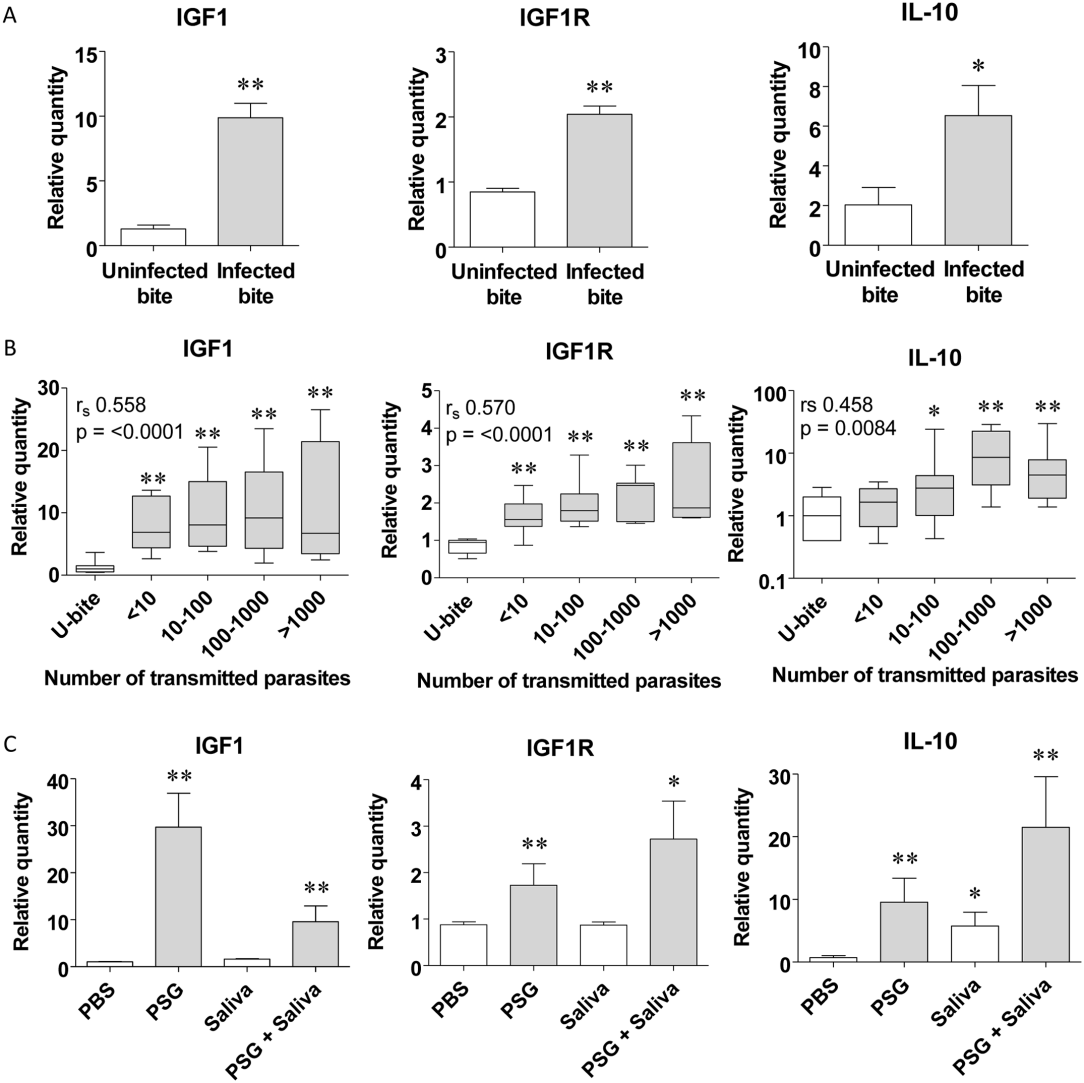
PSG enhances IGF1 expression in skin following an infected fly bite and enhances macrophage alternative activation. A) IGF1, IGF1R and IL-10 expression in BALB/c mouse ears 6 hours post-bite from single uninfected or *L*. *mexicana*-infected *Lu*. *longipalpis* sand flies (Day 8 post-infection), one bite per ear (12 uninfected bites, 35 infected bites). B) IGF1, IGF1R and IL-10 expression in bitten ears in relation to the dose of transmitted parasites, determined from *Leishmania ssrRNA* expression in ears. C) IGF1, IGF1R and IL-10 expression in BALB/c ears 6 hours following intradermal injection of with PBS, 0.5 μg *L*. *mexicana* PSG, 0.5 μg *Lu*. *longipalpis* saliva or PSG and saliva combined. Data is pooled from duplicate experiments with 12 mice per group. Relative expression was normalised to the housekeeping genes *nono* and *l19* and is presented as the mean ±SD (*: p<0.05, **: p<0.005 by Mann Whitney *t*-test).

Having confirmed a role for IGF1-signalling for natural *Leishmania* infection by sand fly bite, we next dissected the contribution made by the various components of transmission: parasites, PSG and sand fly saliva. During transmission, infected sand flies generate a unique wound to a very specific compartment of skin that is difficult to replicate using needles. Using uninfected and *L*. *mexicana*-infected sand flies (with mature, transmissible infections: average ± SD, 3 x 10^4^ ± 1.63 x 10^4^ promastigotes; 58% ± 9% metacyclics per gut), we compared dermal IGF1 expression in response to individual sand fly bites. In this way, each bite received the same wound and deposition of saliva yet the infected bites would additionally receive parasites and PSG. RTqPCR of bitten skin revealed that IGF1, IGF1R and IL-10 expression were significantly higher following infected sand fly bites compared to uninfected bites (IGF1 and IGF1R: p <0.001; IL-10: p <0.05) (Fig 7A). Further analysis of the infected bites by RTqPCR to determine the number of *L*. *mexicana* parasites transmitted (<10 to >10^3^) revealed an association between the dose of parasites delivered per bite with IGF1, IGF1R and IL-10 expression (Fig 7B). Using the *L*. *mexicana-Lu*. *longipalpis* model of natural infection we previously demonstrated a positive relationship between the infective dose and amount of egested PSG [12], which is consistent with one of the main functions of the PSG plug—to block the anterior sand fly midgut for parasite regurgitation. Collectively, this points toward PSG as the main component of natural *Leishmania* infection able to manipulate IGF1 expression and signalling at the site of transmission. To test this, we intradermally injected PSG, saliva and a mixture of PSG and saliva without *Leishmania* into BALB/c ears and found that PSG was the only component of transmission could increase local IGF1, IGF1R and IL-10 expression 6 hours later (Fig 7C: 28-, 2- and 13-fold increase respectively, p <0.005). In contrast, sand fly saliva was only able to moderately enhance IL-10 expression. Furthermore, the combination of PSG with sand fly saliva was still capable of promoting IGF1, IGF1R and IL-10 expression. These data show that regurgitated PSG manipulates IGF1-signalling at the site of transmission.

**Fig 8:**
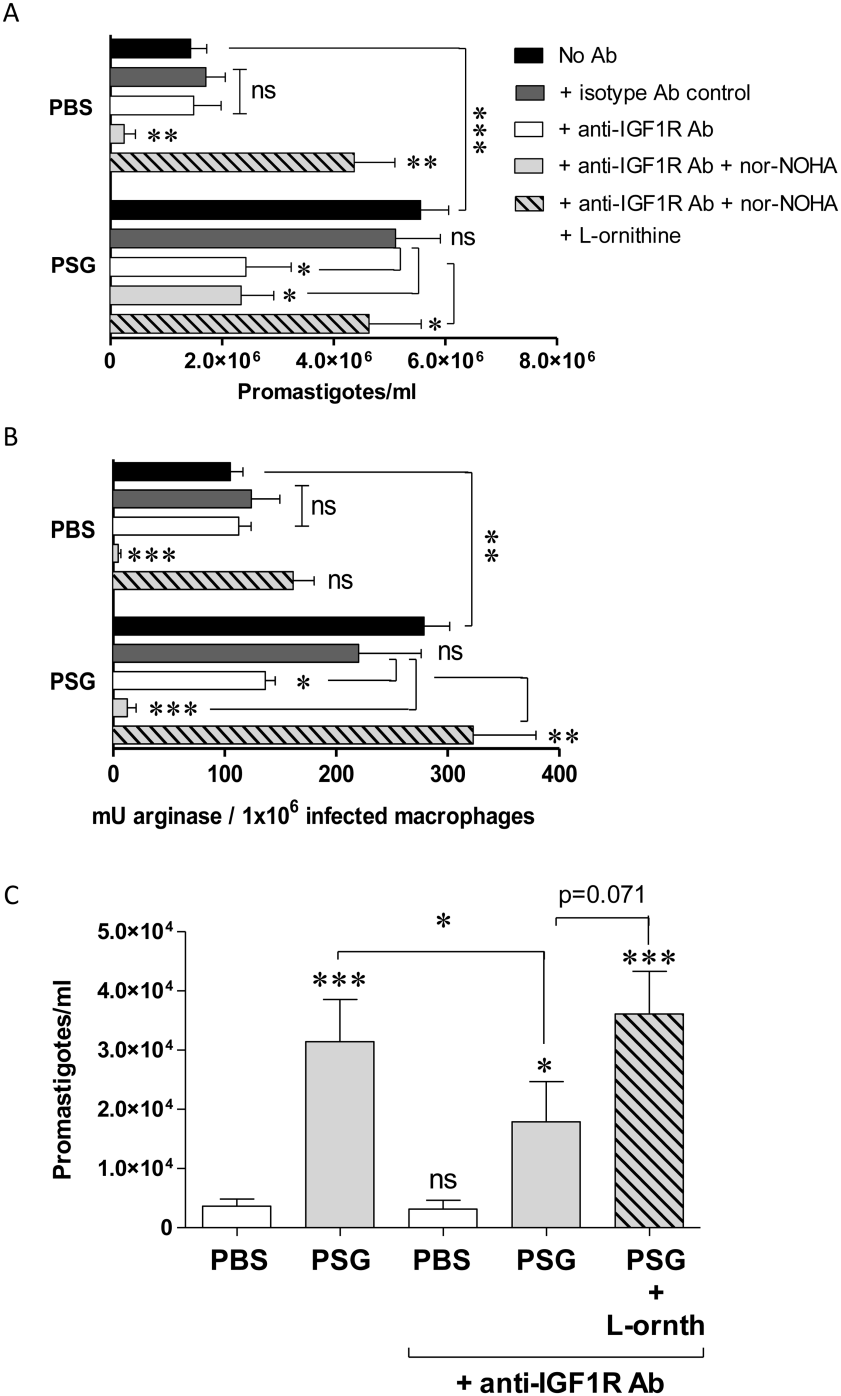
PSG manipulates macrophage arginases to promote intracellular *Leishmania* growth in vitro and in vivo via IGF1 signalling. A) Viable amastigote burdens of macrophages infected *in vitro* was determined by transformation assay of amastigotes into promastigotes liberated from 5 x 10^6^ BALB/c BMMΦ. Macrophages were infected with *L*. *mexicana* metacyclic promastigotes (MOI 1:1) in the presence of ±1 μg *L*. *mexicana* PSG; ±1:100 anti-IGF1R Ab or isotype control; ±100 μM nor-NOHA; ±100 μM L-ornithine for 48 hours. B) Arginase activity of duplicate infected macrophages from (A). C) Parasite burden of BALB/c dermal air-pouches infected with 1x10^6^ *L*. *mexicana* metacyclic promastigotes ±1 μg PSG, ±1:50 anti-IGF1R Ab; ±100 μg L-ornithine for 48 hours. Macrophages were recovered from infected air-pouches and separated by adherence to plastic. Data is representative of triplicate experiments (A&B) or pooled from duplicate experiments (C, 8 mice per group). Unless they are linked with a bar all test groups are compared to their relevant saline control; (ns, not significant p>0.05; *: p<0.05; **: p<0.005; ***: p<0.0005 by Mann Whitney t-test).

PSG can manipulate host macrophage arginase activity to promote the intracellular growth of *L*. *mexicana* [12]. To test the role of PSG-IGF1-signalling for macrophage alternative activation we repeated our experiments using *in vitro* BMMΦ and assessed the role of PSG and IGF1 on parasite viability and host cell arginase activity (Fig 8A & 8B). To determine parasite viability amastigotes were liberated from infected BMMΦ and allowed to transform into flagellated promastigotes *in vitro*–mimicking their onward transmission to sand flies. Similar to our previous findings [12], we found that PSG could promote the survival and growth of amastigotes by enhancing the arginase activity of BMMΦ, which was sensitive to the host-specific arginase inhibitor nor-NOHA. Similarly, the presence of a monoclonal antibody to the IGF1R reduced macrophage infection and arginase activity in PSG-treated cells but not in the saline-treated or isotype antibody controls. Moreover, this could be partially restored by adding L-ornithine—the product of arginase activity on its substrate L-arginine; thereby bypassing arginase for the biosynthesis of polyamines. This indicates that IGF1-blockade affected the macrophage or parasite’s polyamine biosynthetic pathways, reducing the benefit of PSG to amastigotes growth by an average of 72% (p = 0.026). These data show that PSG potentiates host cell activation and polyamine metabolism through IGF1-signalling.

Lastly, to demonstrate that higher IGF1 expression by macrophages exposed to PSG and IGF1-signalling exacerbated *Leishmania* infections *in vivo* we infected dermal air-pouches with 10^6^ *L*. *mexicana* metacyclic promastigotes on BALB/c mice treated with or without anti-IGF1R Ab (Fig 8C). Transformation and growth of amastigotes (as promastigotes) obtained from the infected macrophages recruited to and recovered from the air-pouches revealed that parasite burdens were 8.6-fold higher in those infections receiving PSG without antibody (p = 0.0003), confirming the exacerbatory role of PSG for macrophage infection *in vivo* in skin. In contrast, blockade of IGF1-signalling reduced the exacerbating effect of PSG by 36%, which could be restored with L-ornithine supplementation. Collectively, these data demonstrate the importance of IGF1 for the *Leishmania*-enhancing properties of PSG in skin shortly after transmission—in order to benefit *Leishmania* infection and onward transmission.

## Discussion

In this study we uncover the early interactions between PSG and the mammalian host following *Leishmania* transmission and reveal that it is intimately involved in manipulating the wound healing process that follows the bite of the sand fly vector. We show that PSG promotes both *Leishmania* infection and wound repair through a common mechanism—IGF1-signalling; an important contributor to the normal wound healing process of the body. Through enhanced IGF1-signalling PSG promotes the alternative activation of macrophages recruited to the site of infection and increases their arginase levels. In turn, this increases polyamine biosynthesis that favours the intracellular survival and growth of *Leishmania* and provides the building blocks for collagen synthesis, which accelerates wound repair.

*Leishmania* transmission is a complex event involving contributions made by the unique wound created by the sand fly bite, the vector’s saliva, the egested parasites, parasite exosomes and the regurgitated proteophosphoglycans that make the PSG. The arthropod bite will cause cell damage and injury to blood vessels. In response, blood vessels constrict to reduce blood loss, platelets aggregate and the clotting and complement cascades are activated to form a hemostatic clot. Interestingly, PSG did not appear to influence this early phase of the wound response, suggesting that the close co-evolution between *Leishmania* and their sand fly vectors has allowed the parasite to rely entirely on the vector’s saliva to manipulate hemostasis for bloodfeeding [66]. Clot formation is closely followed by the release of chemoattractants and chemokines from activated platelets to recruit many cell types to the wound bed, including monocytes/macrophages and neutrophils. Mice that received PSG displayed an enhanced pro-inflammatory and chemokine response 4 hours post-wound—resulting in elevated expression of the inflammatory cytokines IL-1β, IL-6 and TNF, and the chemokines CXCL2, CCL4 accompanied by moderate increases in CCL3 and CCL2. A similar expression profile (S3 Fig) and protein production (S4 Fig) was seen for an early (6 and 24h, respectively) *L*. *mexicana* infection in the presence of PSG. In fresh wounds, a strong pro-inflammatory response is required to sterilise the damaged tissue of potentially pathogenic bacteria that are introduced during trauma though recruitment of neutrophils and classical activation of macrophages. Such a pro-inflammatory milieu could be unfavourable to *Leishmania* survival. Moreover, the adaptive immune response is greatly influenced by the local microenvironment during first few hours post-infection [67–71] and may further impact *Leishmania* infection. Despite this, we could detect an 8-fold increase in *Leishmania ssrRNA* expression in ears co-infected with PSG by as early as 6 hours post-infection, suggesting that *L*. *mexicana* survival (but not necessarily replication) benefitted from the presence of gel. Similarly, following 48 hours of infection, PSG promoted *L*. *mexicana* growth in dermal air-pouches that were conditioned with the pro-inflammatory mediators IFN-γ and TNFα to induce a type1 immune environment [12]. The longer-term benefit of PSG to *Leishmania* growth is clear, as previous studies have repeatedly shown that PSG can promote chronic infections in both susceptible and resistant mouse strains, for a wide range of parasite doses and parasite species [6,12,13]. Collectively, these data indicate that PSG affords *Leishmania* protection from a hostile pro-inflammatory environment and may direct its action towards macrophage alternative activation, since these cells are important in dampening the early pro-inflammatory response in a wound and orchestrate wound closure and re-epithelialisation [72].

The ability of *Leishmania* to skew their host macrophages towards alternative activation is emerging as an important survival strategy following vector transmission. Previously, we found that *in vivo Leishmania* infection of macrophages recruited to air-pouches exposed to inducers of iNOS were less efficient at killing when exposed to PSG by expressing higher arginase levels [12]. Affymetrix analysis revealed that AAMΦ/M2-associated transcription factors STAT6 (FC: +2.48, p <0.0005), STAT3 (FC: +6.14, p <0.0005) and Sp1 (FC: +3.5, p <0.0005), but not CAMΦ/M1-associated transcription factors STAT1 (FC: -1.70, p <0.005) or STAT5 (FC: -1.87, p <0.005), were up-regulated in response to PSG in skin by 6 hours post-infection, and was reproduced *in vitro* with BMMΦ (Fig 4C). This supports the idea that macrophages can be induced to express Arg1 through multiple means, including cytokine-dependent, STAT6 pathways as well as TLR-dependent, STAT3 pathways [73,74]. As a result, this feature of macrophage alternative activation may allow PSG to generate a limited pro-inflammatory response in skin that can both kick-start an efficient wound healing response to the vector’s bite and enhance the alternative activation of macrophages recruited to the wound. Therefore, the ability of PSG to promote *Leishmania* infection of CAMΦ or in skin preconditioned with IFN-γ and TNFα [12] appears to help parasites to survive the intense period of inflammation and wound sterilisation immediately following the sand fly bite. Counter-intuitively, this may be beneficial to the establishing infection by reducing the competition for host cells and resources from bacteria potentially co-inoculated by the sand fly bite. Despite being considered a prototypic M2 myeloid cell marker of alternative activation, Arg1 can also be expressed by CAMΦ in a STAT3-dependent pathway activated by IL-10, IL-6 or macrophage colony stimulating factor (M-CSF) [74]. In support of this we show that PSG up-regulates the early expression of Arg1, IL-10, IL-6 and IGF1 during cutaneous *L*. *mexicana* infection *in vivo* and when co-incubated with macrophages *in vitro*. Further, other genes involved in macrophage alternative activation, including the murine mannose receptor (CD206), resistin-like alpha (Fizz1), Ym1, Fn1, Odc1 and IGF1R are increased in response to PSG [12, this study]; indicating that PSG may induce Arg1 by multiple pathways, including both STAT3 and STAT6. In a hamster model of visceral leishmaniasis (VL), Osorio and colleagues showed that *Leishmania donovani* infected macrophages displayed enhanced STAT6 and STAT3 signalling in response to the growth factors FGF2 and IGF1, further enhanced by co-stimulation with IL-4 or IL-10 [75]. They put forward that a parasite-derived factor could trigger the FGF2 and IGF1 receptors during VL. Our results indicate that a component of the PSG promotes IGF1-signalling in a similar way by enhancing receptor and ligand expression in dermal macrophages during the early phase of infection when macrophage polarisation can significantly influence the course of infection. Although the present study did not focus on the identity of the PSG’s active ingredient/s, we have previously seen that purified fPPG can enhance cutaneous *L*. *mexicana* infection in BALB/c mice [6], supporting the ability of *Leishmania* PPGs to direct these related processes. In the Osorio-VL model PSG or fPPG was absent, however, within infected phagolysosomes of macrophages amastigotes secrete a complex proteophosphoglycan (aPPG) which can reach very high concentrations (40–100 mg/g in cutaneous lesions of *L*. *mexicana*) and has biochemical similarity to PPGs in the PSG [14]. Therefore, we speculate that aPPG may be the parasite-factor driving the FGF2/IGF1-STAT6 induction of Arg1 in VL, or potentially in established cutaneous lesions. If so, it will show that *Leishmania* PPGs have the ability to extend their manipulation of host macrophage alternative activation immediately following transmission (as fPPG in PSG) into the chronic phase of cutaneous or visceral infection (as aPPG).

Arguably, the most important contribution of AAMΦ in wound healing is the expression of growth factors, including IGF1, FGF2, VEGF and TGFβ, as these factors promote angiogenesis and activate fibroblasts. Here we show that PSG could also enhance the expression of IGF1 and its receptor in skin and promote the proliferation and migration of a fibroblast cell line to resolve an *in vitro* scratch wound. Arg1 expression is not restricted to STAT6 phosphorylation and can be modulated by a number of growth factor receptor pathways [76]. To our knowledge, the only host growth factors known to have a direct influence over *Leishmania* infection and Arg1 expression are IGF1 and FGF2 [63–65, 75]. Studies involving a wide range of *Leishmania* species have shown that IGF1 benefits cutaneous *Leishmania* infection and that this is dependent on macrophage arginase. Extending our previous findings [12], PSG enhanced the expression of Arg1 in the mouse dermis by promoting both IGF1 production and signalling. As discussed above, infected macrophage Arg1 expression is driven by FGF2 and IGF1, via STAT6, and promoted by co-stimulation with IL-4 [75]. Therefore, our results indicate that a similar mechanism operates in skin shortly following transmission, amplified by the presence of regurgitated PSG from the infected sand fly. Macrophages stimulated via the IL-4 receptor (IL-4Rα) have also been shown to express IGF1 [76], however, we could not detect any direct modulation of IL-4, IL-13 or their receptor IL-4Rα by PSG and PSG did not enhance CL in IL-4Rα-deficient mice; suggesting that PSG signals via different cytokine pathways to promote IGF1 secretion. Interestingly, our Affymetrix study also showed canonical pathways involving mammalian target of rapamycin (mTOR) signalling, hypoxia and protein ubiquitination to be activated by PSG in skin higher than IGF1 signalling (Table 1 and S1 Table). mTOR is an important regulator of mammalian metabolism and physiology and central sensor of cellular injury, regulated by both hypoxia and ubiquitination, amongst others. Notably, mTOR complex 2 (mTORC2) exhibits tryposine protein kinase activity which phosphorylates the IGF1R, leading to its full activation [77]. This indicates that there is a potential synergy between PSG-induced mTOR and IGF1 signalling and that PSG may manipulate a larger network of cellular processes, involving nutrient-, oxygen- and energy-sensing pathways.

A close association between injury and the induction of type 2 (anti-inflammatory) immunity is required to limit excessive inflammation. In this regard, IGF1 has been shown to be essential to limit damage created by helminth parasites as they transiently migrate through the lung by promoting macrophage alternative activation [78]. Alternatively activated macrophages dampen inflammation through a variety of mechanisms, including cytokines, receptors and enzymes. During infection with the helminth parasite *Nippostrongylus braziliensis*, AAMΦ-induced IL-10 and IGF1 suppress the pro-inflammatory Th17 and neutrophil response [19,20]. Neutrophils are an important cell type for *Leishmania* which respond to dermal injury and rapidly infiltrate a sand fly bite and the injection of PSG [12,27]. Intravital imaging of fluorescent neutrophils have shown that these are probably the first cells to be parasitized by *Leishmania* following transmission and the presence of neutrophils during needle- or sand fly-delivered infections benefitted the survival of *L*. *major* [26]. It is thought that neutrophils provide temporary shelter for *Leishmania* during the pro-inflammatory phase of the wound response following transmission and allow silent infection of macrophages when they ingest the apoptotic infected neutrophils—the “Trojan Horse” theory of infection [27, 79,80]. The importance of neutrophils for leishmaniasis is underlined by the fact that vaccine efficacy is boosted in the absence of neutrophils [81]. More recently, exosomes released by *Leishmania* promastigotes inside the sand fly midgut are a newly identified component of sand fly transmission, which can potentiate parasite infection in mice [82]. Notably, *Leishmania* exosomes induced a strong IL-17a response in skin and we can also detect an increase in IL-17a levels in response to the injection of PSG (S5 Fig). This suggests that PSG may act as a carrier for *Leishmania* exosomes to ensure their transmission and warrants further investigation. During such studies, it will be pertinent to examine the role of PSG and exosomes on Th17 biology since this is a significant driver of inflammation, neutrophil recruitment and susceptibility to leishmaniasis [83–87].

Following the inoculation of PSG into skin there was also a sharp increase in IL-6 expression. IL-6 is a pluripotent cytokine that has well characterised pro-inflammatory properties but can act as a regulator of macrophage alternative activation by inducing the expression of the IL-4 receptor in a STAT3-dependent manner [88]. In our experiments, we could not detect any modulation of the expression of IL-4Rα by PSG, suggesting that IL-6 is unlikely to participate directly in the action of PSG. However, an indirect role for IL-6 may come from its ability to stimulate the expression and secretion of IL-10 from macrophages [89], potentially amplifying the arginase activation in macrophages through the IL-10/IGF1 axis. In support of this, we found that PSG and infected sand fly bites promote the expression of IL-10 in skin.

Here we provide the first evidence that the production of IGF1 during natural *Leishmania* infection, i.e. in response to regurgitated PSG from infected sand fly bites, is important in the wound healing response. We show that through PSG, *Leishmania* have evolved to take advantage of the wound created by the vector bite and the host’s need to control excessive collateral tissue damage during the initial pro-inflammatory phase of wound healing. To do this PSG exaggerates the inflammatory phase of the early wound response to induce IGF1-signalling and IGF1-dependent expression of Arg1 in macrophages. As a result, the increased arginase-mediated L-arginine catabolism of parasitized AAMΦ recruited to heal the exaggerated wound promote *Leishmania* infection in skin. In a sterile wound, PSG can potently accelerate wound resolution and we speculate that PSG, or defined components thereof, could be exploited as a future wound therapeutic.

## Supporting information

S1 Table Top 168 canonical pathways influenced by PSG in skin. Results of IPA of 5,312 genes of known function modulated by PSG (cut off ≥ FC5 at 5% FDR).
(DOCX)

S2 Table Gene Ontology enrichment analysis of genes influenced by PSG in skin. (DOCX)

S3 Table RTqPCR primer sequences and melting temperatures used in this study. The genes used to validate gene expression in the microarray study (Fig 1E) are denoted with an asterisk.
(DOCX)

S1 Fig PSG does not influence scratch wound healing to cultured keratinocytes. Monolayers of Kera308 keratinocytes were scratched in the presence of culture media supplemented with or without 0.5 μg/ml *L*. *mexicana* PSG. Positive controls were treated with 10 μg/ml TGFβ2 and 10 μg/ml EGF. A) At 0, 12 and 24 hours post-wound, photomicrographs were taken and scratch closure was determined from using ImageJ. Statistical analyses were performed between Media vs. PSG at each time point. Each in condition was performed in quadruplicate, data is pooled from 3 experiments. Average wound closure ±SEM is shown (*: p<0.05; **: p<0.005; ***: p<0.0005 by Mann Whitney *t*-test).
(TIF)

S2 Fig PSG promotes JAK-STAT signalling. STAT3-Luciferase reporter HeLa cells were exposed to 1 μg PSG ± 1:100 anti-IGF1R Ab for 24 hours. Positive controls were treated with 1 ng/ml and 10 ng/ml Oncostatin M for 8 hours. Luciferase expression was recorded with a luminomter. Results are from a representative experiment. Relative light intensity is presented as the mean ±SD of 4 wells per group. (*: p<0.05, **: p<0.005 by Mann Whitney t-test).
(TIF)

S3 Fig PSG augments the wound response during *L*. *mexicana* infection. Ears of BALB/c mice were intra-dermally inoculated with 1 x 10^3^ *L*. *mexicana* metacyclic promastigotes ±0.5 μg *L*. *mexicana* PSG or PBS. A-C) Six hours post-infection ears were measured for transcripts involved in the inflammation and cell proliferation phases of wound healing by real-time quantitative PCR. A) Chemokines: CCL2, CCL3, CCL4 and CXCL2, B) pro-inflammatory-modulating cytokines: IL-1α, IL-1β, IL-6, IL-10 and TNFα, and C) epidermal growth factors and receptors: EGF, IGF1, EGFR, IGF1R and FGFR2. Relative expression was normalised to the housekeeping genes *nono* and *l19* and is presented as the mean ±SD with 9–12 mice per group. (*: p<0.05, **: p<0.005 by Mann Whitney *t*-test).
(TIF)

S4 Fig PSG augments the production of chemokines and cytokines involved in the early wound response during *L*. *mexicana* infection. Ears of BALB/c mice were intra-dermally inoculated with 1 x 10^3^ *L*. *mexicana* metacyclic promastigotes ±0.5 μg *L*. *mexicana* PSG or PBS. A and B) Twenty four hours post-infection ears were measured for proteins involved in the inflammation phase of wound healing by real-time quantitative PCR. A) Chemokines: CCL2, CCL3, CCL4 and CXCL2 and B) pro-inflammatory-modulating cytokines: IL-1α, IL-1β, IL-6, IL-10 and TNFα. Protein levels were determined by Luminex from the ear lysates used in S3 Fig, and is presented as the mean ±SD with 9–12 mice per group. (*: p<0.05 by Mann Whitney *t*-test).
(TIF)

S5 Fig PSG promotes IL-17a production in skin at the bite site 6 hours post-infection. Ears of BALB/c mice were intra-dermally inoculated with 1 x 10^3^ *L*. *mexicana* metacyclic promastigotes ±0.5 μg/ml *L*. *mexicana* PSG. IL-17a production was determined from whole cell lysates using Luminex. Data is representative duplicate experiments (*: p<0.05 by Mann Whitney *t*-test).
(TIF)
