# Supplementary material for: Leishmania proteophosphoglycans regurgitated from infected sand flies accelerate dermal wound repair and exacerbate leishmaniasis via insulin-like growth factor 1-dependent signalling
Source: PLoS Pathog. 2018 Jan 19;14(1):e1006794. doi: 10.1371/journal.ppat.1006794 (PMC5792026; doi:10.1371/journal.ppat.1006794)
Supplement: S1 Table — Results of IPA of 5,312 genes of known function modulated by PSG (cut off ≥ FC5 at 5% FDR). (DOCX) [file ppat.1006794.s001.docx]

| Supplementary Table 1, Giraud E et al. | | | | |
| --- | --- | --- | --- | --- |
|  | **Ingenuity Canonical Pathway** | **-log(p-value)** | **pvalue** | **%** |
| 1 | **EIF2 Signaling** | 31,80 | 1,58E-32 | 27,6 |
| 2 | **Protein Ubiquitination Pathway** | 20,40 | 3,98E-21 | 20,4 |
| 3 | **Regulation of eIF4 and p70S6K Signaling** | 19,90 | 1,26E-20 | 23,5 |
| 4 | **Oxidative Phosphorylation** | 17,30 | 5,01E-18 | 21,9 |
| 5 | **mTOR Signaling** | 15,10 | 7,94E-16 | 19,4 |
| 6 | **Mitochondrial Dysfunction** | 10,30 | 5,01E-11 | 15,4 |
| 7 | **Hypoxia Signaling in the Cardiovascular System** | 8,28 | 5,25E-09 | 25,0 |
| 8 | **PI3K/AKT Signaling** | 7,90 | 1,26E-08 | 16,4 |
| 9 | **Citrate Cycle** | 6,08 | 8,32E-07 | 17,5 |
| 10 | **Telomerase Signaling** | 5,79 | 1,62E-06 | 17,5 |
| 11 | **ERK/MAPK Signaling** | 5,66 | 2,19E-06 | 12,7 |
| 12 | **Ephrin Receptor Signaling** | 5,25 | 5,62E-06 | 12,0 |
| 13 | **Breast Cancer Regulation by Stathmin1** | 4,99 | 1,02E-05 | 11,9 |
| 14 | **Protein Kinase A Signaling** | 4,99 | 1,02E-05 | 10,3 |
| 15 | **Phenylalanine Metabolism** | 4,84 | 1,45E-05 | 9,0 |
| 16 | **Role of NFAT in Regulation of the Immune Response** | 4,65 | 2,24E-05 | 11,0 |
| 17 | **IGF-1 Signaling** | 4,60 | 2,51E-05 | 15,0 |
| 18 | **Production of Nitric Oxide and Reactive Oxygen Species in Macrophages** | 4,58 | 2,63E-05 | 11,0 |
| 19 | **Signaling by Rho Family GTPases** | 4,54 | 2,88E-05 | 10,5 |
| 20 | **Purine Metabolism** | 4,54 | 2,88E-05 | 7,5 |
| 21 | **Ubiquinone Biosynthesis** | 4,49 | 3,24E-05 | 10,5 |
| 22 | **Prostate Cancer Signaling** | 4,44 | 3,63E-05 | 14,4 |
| 23 | **RhoA Signaling** | 4,44 | 3,63E-05 | 14,9 |
| 24 | **Butanoate Metabolism** | 4,41 | 3,89E-05 | 9,3 |
| 25 | **Cell Cycle Regulation by BTG Family Proteins** | 4,33 | 4,68E-05 | 25,0 |
| 26 | **ERK5 Signaling** | 4,33 | 4,68E-05 | 18,8 |
| 27 | **Actin Cytoskeleton Signaling** | 4,25 | 5,62E-05 | 10,5 |
| 28 | **fMLP Signaling in Neutrophils** | 4,20 | 6,31E-05 | 12,5 |
| 29 | **Tight Junction Signaling** | 4,19 | 6,46E-05 | 12,2 |
| 30 | **NRF2-mediated Oxidative Stress Response** | 4,11 | 7,76E-05 | 11,5 |
| 31 | **ILK Signaling** | 4,11 | 7,76E-05 | 11,4 |
| 32 | **Clathrin-mediated Endocytosis Signaling** | 3,96 | 1,10E-04 | 11,3 |
| 33 | **Valine, Leucine and Isoleucine Degradation** | 3,90 | 1,26E-04 | 11,1 |
| 34 | **Regulation of Actin-based Motility by Rho** | 3,71 | 1,95E-04 | 14,3 |
| 35 | **Integrin Signaling** | 3,50 | 3,16E-04 | 10,5 |
| 36 | **CDK5 Signaling** | 3,49 | 3,24E-04 | 13,8 |
| 37 | **PI3K Signaling in B Lymphocytes** | 3,46 | 3,47E-04 | 11,6 |
| 38 | **p70S6K Signaling** | 3,39 | 4,07E-04 | 12,0 |
| 39 | **RhoGDI Signaling** | 3,38 | 4,17E-04 | 10,0 |
| 40 | **Phospholipase C Signaling** | 3,36 | 4,37E-04 | 9,2 |
| 41 | **Virus Entry via Endocytic Pathways** | 3,33 | 4,68E-04 | 13,0 |
| 42 | **Dopamine-DARPP32 Feedback in cAMP Signaling** | 3,30 | 5,01E-04 | 10,2 |
| 43 | **CCR3 Signaling in Eosinophils** | 3,29 | 5,13E-04 | 11,9 |
| 44 | **p53 Signaling** | 3,23 | 5,89E-04 | 13,5 |
| 45 | **Gα12/13 Signaling** | 3,20 | 6,31E-04 | 11,7 |
| 46 | **Fcγ Receptor-mediated Phagocytosis in Macrophages and Monocytes** | 3,13 | 7,41E-04 | 12,7 |
| 47 | **B Cell Receptor Signaling** | 3,07 | 8,51E-04 | 10,9 |
| 48 | **Pyruvate Metabolism** | 3,04 | 9,12E-04 | 7,4 |
| 49 | **Methane Metabolism** | 2,97 | 1,07E-03 | 7,5 |
| 50 | **Glucocorticoid Receptor Signaling** | 2,93 | 1,17E-03 | 8,5 |
| 51 | **CD28 Signaling in T Helper Cells** | 2,85 | 1,41E-03 | 10,6 |
| 52 | **Cardiac Hypertrophy Signaling** | 2,85 | 1,41E-03 | 9,0 |
| 53 | **Neuregulin Signaling** | 2,80 | 1,58E-03 | 11,8 |
| 54 | **Ceramide Signaling** | 2,80 | 1,58E-03 | 12,6 |
| 55 | **Insulin Receptor Signaling** | 2,77 | 1,70E-03 | 10,7 |
| 56 | **CXCR4 Signaling** | 2,76 | 1,74E-03 | 10,1 |
| 57 | **Calcium Signaling** | 2,72 | 1,91E-03 | 8,7 |
| 58 | **Cdc42 Signaling** | 2,70 | 2,00E-03 | 8,3 |
| 59 | **Rac Signaling** | 2,70 | 2,00E-03 | 10,6 |
| 60 | **NGF Signaling** | 2,66 | 2,19E-03 | 11,2 |
| 61 | **Antigen Presentation Pathway** | 2,63 | 2,34E-03 | 14,0 |
| 62 | **Synaptic Long Term Potentiation** | 2,62 | 2,40E-03 | 11,4 |
| 63 | **Aldosterone Signaling in Epithelial Cells** | 2,61 | 2,45E-03 | 9,8 |
| 64 | **Fatty Acid Elongation in Mitochondria** | 2,61 | 2,45E-03 | 10,6 |
| 65 | **Huntington's Disease Signaling** | 2,60 | 2,51E-03 | 8,8 |
| 66 | **Mitotic Roles of Polo-Like Kinase** | 2,57 | 2,69E-03 | 13,8 |
| 67 | **Cardiac β-adrenergic Signaling** | 2,56 | 2,75E-03 | 9,7 |
| 68 | **Nitric Oxide Signaling in the Cardiovascular System** | 2,56 | 2,75E-03 | 10,0 |
| 69 | **P2Y Purigenic Receptor Signaling Pathway** | 2,55 | 2,82E-03 | 10,0 |
| 70 | **Thrombin Signaling** | 2,47 | 3,39E-03 | 9,2 |
| 71 | **Estrogen-Dependent Breast Cancer Signaling** | 2,46 | 3,47E-03 | 12,9 |
| 72 | **Androgen Signaling** | 2,44 | 3,63E-03 | 9,0 |
| 73 | **CTLA4 Signaling in Cytotoxic T Lymphocytes** | 2,40 | 3,98E-03 | 11,2 |
| 74 | **HGF Signaling** | 2,39 | 4,07E-03 | 11,4 |
| 75 | **Dopamine Receptor Signaling** | 2,37 | 4,27E-03 | 10,5 |
| 76 | **α-Adrenergic Signaling** | 2,36 | 4,37E-03 | 10,4 |
| 77 | **PPARα/RXRα Activation** | 2,32 | 4,79E-03 | 9,0 |
| 78 | **FAK Signaling** | 2,32 | 4,79E-03 | 10,8 |
| 79 | **SAPK/JNK Signaling** | 2,32 | 4,79E-03 | 10,8 |
| 80 | **Circadian Rhythm Signaling** | 2,31 | 4,90E-03 | 17,1 |
| 81 | **Polyamine Regulation in Colon Cancer** | 2,31 | 4,90E-03 | 17,2 |
| 82 | **Apoptosis Signaling** | 2,28 | 5,25E-03 | 11,5 |
| 83 | **CREB Signaling in Neurons** | 2,27 | 5,37E-03 | 8,4 |
| 84 | **Sertoli Cell-Sertoli Cell Junction Signaling** | 2,27 | 5,37E-03 | 8,5 |
| 85 | **Cyclins and Cell Cycle Regulation** | 2,25 | 5,62E-03 | 11,2 |
| 86 | **Glycerolipid Metabolism** | 2,24 | 5,75E-03 | 7,1 |
| 87 | **RAR Activation** | 2,24 | 5,75E-03 | 9,1 |
| 88 | **Actin Nucleation by ARP-WASP Complex** | 2,17 | 6,76E-03 | 12,1 |
| 89 | **Renal Cell Carcinoma Signaling** | 2,13 | 7,41E-03 | 12,2 |
| 90 | **Role of Tissue Factor in Cancer** | 2,11 | 7,76E-03 | 10,5 |
| 91 | **Arginine and Proline Metabolism** | 2,09 | 8,13E-03 | 5,1 |
| 92 | **Aryl Hydrocarbon Receptor Signaling** | 2,08 | 8,32E-03 | 8,8 |
| 93 | **Propanoate Metabolism** | 2,07 | 8,51E-03 | 6,6 |
| 94 | **EGF Signaling** | 2,07 | 8,51E-03 | 13,5 |
| 95 | **IL-6 Signaling** | 2,06 | 8,71E-03 | 11,0 |
| 96 | **Caveolar-mediated Endocytosis Signaling** | 2,01 | 9,77E-03 | 10,6 |
| 97 | **Stilbene, Coumarine and Lignin Biosynthesis** | 1,98 | 1,05E-02 | 5,4 |
| 98 | **Antiproliferative Role of Somatostatin Receptor 2** | 1,98 | 1,05E-02 | 11,3 |
| 99 | **Gap Junction Signaling** | 1,98 | 1,05E-02 | 8,2 |
| 100 | **FGF Signaling** | 1,98 | 1,05E-02 | 11,1 |
| 101 | **VEGF Signaling** | 1,98 | 1,05E-02 | 10,1 |
| 102 | **PDGF Signaling** | 1,97 | 1,07E-02 | 11,4 |
| 103 | **CNTF Signaling** | 1,97 | 1,07E-02 | 12,7 |
| 104 | **Lysine Degradation** | 1,94 | 1,15E-02 | 5,8 |
| 105 | **LPS-stimulated MAPK Signaling** | 1,93 | 1,17E-02 | 11,0 |
| 106 | **Mechanisms of Viral Exit from Host Cells** | 1,93 | 1,17E-02 | 13,3 |
| 107 | **Atherosclerosis Signaling** | 1,92 | 1,20E-02 | 9,2 |
| 108 | **Wnt/β-catenin Signaling** | 1,92 | 1,20E-02 | 9,2 |
| 109 | **G Beta Gamma Signaling** | 1,91 | 1,23E-02 | 8,6 |
| 110 | **iCOS-iCOSL Signaling in T Helper Cells** | 1,90 | 1,26E-02 | 9,0 |
| 111 | **CCR5 Signaling in Macrophages** | 1,89 | 1,29E-02 | 8,5 |
| 112 | **Relaxin Signaling** | 1,87 | 1,35E-02 | 8,2 |
| 113 | **Glycolysis/Gluconeogenesis** | 1,82 | 1,51E-02 | 6,8 |
| 114 | **AMPK Signaling** | 1,82 | 1,51E-02 | 7,7 |
| 115 | **Non-Small Cell Lung Cancer Signaling** | 1,77 | 1,70E-02 | 10,1 |
| 116 | **IL-4 Signaling** | 1,77 | 1,70E-02 | 11,0 |
| 117 | **Neurotrophin/TRK Signaling** | 1,77 | 1,70E-02 | 10,4 |
| 118 | **IL-1 Signaling** | 1,77 | 1,70E-02 | 9,4 |
| 119 | **Synthesis and Degradation of Ketone Bodies** | 1,76 | 1,74E-02 | 15,8 |
| 120 | **IL-2 Signaling** | 1,74 | 1,82E-02 | 12,1 |
| 121 | **Regulation of IL-2 Expression in Activated and Anergic T Lymphocytes** | 1,72 | 1,91E-02 | 10,1 |
| 122 | **Melanoma Signaling** | 1,72 | 1,91E-02 | 13,0 |
| 123 | **Corticotropin Releasing Hormone Signaling** | 1,72 | 1,91E-02 | 8,1 |
| 124 | **PPAR Signaling** | 1,71 | 1,95E-02 | 9,4 |
| 125 | **Dendritic Cell Maturation** | 1,69 | 2,04E-02 | 6,9 |
| 126 | **Fatty Acid Metabolism** | 1,69 | 2,04E-02 | 6,0 |
| 127 | **Chronic Myeloid Leukemia Signaling** | 1,68 | 2,09E-02 | 9,5 |
| 128 | **Germ Cell-Sertoli Cell Junction Signaling** | 1,67 | 2,14E-02 | 8,4 |
| 129 | **IL-10 Signaling** | 1,66 | 2,19E-02 | 10,3 |
| 130 | **Macropinocytosis Signaling** | 1,66 | 2,19E-02 | 10,5 |
| 131 | **Role of NFAT in Cardiac Hypertrophy** | 1,66 | 2,19E-02 | 7,6 |
| 132 | **Calcium-induced T Lymphocyte Apoptosis** | 1,65 | 2,24E-02 | 10,0 |
| 133 | **Lipid Antigen Presentation by CD1** | 1,65 | 2,24E-02 | 13,0 |
| 134 | **T Cell Receptor Signaling** | 1,65 | 2,24E-02 | 9,2 |
| 135 | **Acute Phase Response Signaling** | 1,65 | 2,24E-02 | 8,4 |
| 136 | **IL-8 Signaling** | 1,65 | 2,24E-02 | 7,8 |
| 137 | **IL-17 Signaling** | 1,63 | 2,34E-02 | 10,8 |
| 138 | **Chemokine Signaling** | 1,63 | 2,34E-02 | 11,0 |
| 139 | **Glioma Signaling** | 1,62 | 2,40E-02 | 8,9 |
| 140 | **Myc Mediated Apoptosis Signaling** | 1,61 | 2,45E-02 | 11,5 |
| 141 | **Inhibition of Angiogenesis by TSP1** | 1,61 | 2,45E-02 | 12,8 |
| 142 | **LXR/RXR Activation** | 1,61 | 2,45E-02 | 8,4 |
| 143 | **Xenobiotic Metabolism Signaling** | 1,60 | 2,51E-02 | 6,8 |
| 144 | **Paxillin Signaling** | 1,59 | 2,57E-02 | 8,9 |
| 145 | **FLT3 Signaling in Hematopoietic Progenitor Cells** | 1,59 | 2,57E-02 | 10,8 |
| 146 | **IL-3 Signaling** | 1,56 | 2,75E-02 | 10,8 |
| 147 | **14-3-3-mediated Signaling** | 1,53 | 2,95E-02 | 8,9 |
| 148 | **Sphingolipid Metabolism** | 1,53 | 2,95E-02 | 7,1 |
| 149 | **Oncostatin M Signaling** | 1,51 | 3,09E-02 | 14,3 |
| 150 | **Neuroprotective Role of THOP1 in Alzheimer's Disease** | 1,51 | 3,09E-02 | 9,3 |
| 151 | **Ovarian Cancer Signaling** | 1,48 | 3,31E-02 | 8,5 |
| 152 | **Systemic Lupus Erythematosus Signaling** | 1,47 | 3,39E-02 | 6,2 |
| 153 | **NF-κB Activation by Viruses** | 1,46 | 3,47E-02 | 9,8 |
| 154 | **Amyloid Processing** | 1,45 | 3,55E-02 | 10,7 |
| 155 | **IL-15 Signaling** | 1,43 | 3,72E-02 | 10,4 |
| 156 | **IL-17A Signaling in Airway Cells** | 1,43 | 3,72E-02 | 9,7 |
| 157 | **GM-CSF Signaling** | 1,43 | 3,72E-02 | 10,4 |
| 158 | **Leukocyte Extravasation Signaling** | 1,43 | 3,72E-02 | 7,5 |
| 159 | **IL-12 Signaling and Production in Macrophages** | 1,41 | 3,89E-02 | 7,1 |
| 160 | **Tryptophan Metabolism** | 1,41 | 3,89E-02 | 4,7 |
| 161 | **HER-2 Signaling in Breast Cancer** | 1,40 | 3,98E-02 | 9,9 |
| 162 | **Tyrosine Metabolism** | 1,37 | 4,27E-02 | 3,5 |
| 163 | **Synaptic Long Term Depression** | 1,36 | 4,37E-02 | 7,5 |
| 164 | **Glutathione Metabolism** | 1,34 | 4,57E-02 | 6,5 |
| 165 | **PTEN Signaling** | 1,33 | 4,68E-02 | 8,1 |
| 166 | **Endometrial Cancer Signaling** | 1,30 | 5,01E-02 | 10,5 |
| 167 | **Angiopoietin Signaling** | 1,30 | 5,01E-02 | 9,5 |
| 168 | **Axonal Guidance Signaling** | 1,30 | 5,01E-02 | 6,2 |
