# Supplementary material for: Leishmania proteophosphoglycans regurgitated from infected sand flies accelerate dermal wound repair and exacerbate leishmaniasis via insulin-like growth factor 1-dependent signalling
Source: PLoS Pathog. 2018 Jan 19;14(1):e1006794. doi: 10.1371/journal.ppat.1006794 (PMC5792026; doi:10.1371/journal.ppat.1006794)
Supplement: S2 Table — (DOCX) [file ppat.1006794.s002.docx]

| Supplementary Table 2, Giraud E et al. | |  |  |  |  |
| --- | --- | --- | --- | --- | --- |
| Pathway ID | **Pathways** | | **Size** | **FE** | **P-value** |
| mmu4420097 | **VEGFA-VEGFR2 Pathway** | | 285 | 3.99 | 8,91E-13 |
| mmu194138 | **Signaling by VEGF** | | 292 | 3.89 | 2,25E-12 |
| mmu69613 | **p53-Independent G1/S DNA damage checkpoint** | | 56 | 9.34 | 4,40E-12 |
| mmu69610 | **p53-Independent DNA Damage Response** | | 56 | 9.34 | 4,40E-12 |
| mmu5658442 | **Regulation of RAS by GAPs** | | 70 | 8.12 | 6,29E-12 |
| mmu69541 | **Stabilization of p53** | | 58 | 9.02 | 9,09E-12 |
| mmu350562 | **Regulation of ornithine decarboxylase (ODC)** | | 53 | 9.44 | 1,42E-11 |
| mmu3858494 | **Beta-catenin independent WNT signaling** | | 107 | 6.16 | 4,24E-11 |
| mmu5607764 | **CLEC7A (Dectin-1) signaling** | | 93 | 6.60 | 6,67E-11 |
| mmu5654738 | **Signaling by FGFR2** | | 311 | 3.58 | 9,19E-11 |
| mmu69580 | **p53-Dependent G1/S DNA damage checkpoint** | | 66 | 7.93 | 1,28E-10 |
| mmu69563 | **p53-Dependent G1 DNA Damage Response** | | 66 | 7.93 | 1,28E-10 |
| mmu190236 | **Signaling by FGFR** | | 317 | 3.52 | 1,83E-10 |
| mmu351202 | **Metabolism of polyamines** | | 82 | 6.93 | 2,00E-10 |
| mmu186797 | **Signaling by PDGF** | | 319 | 3.49 | 2,29E-10 |
| mmu195721 | **Signaling by Wnt** | | 221 | 4.12 | 2,83E-10 |
| mmu5621481 | **C-type lectin receptors (CLRs)** | | 116 | 5.69 | 3,05E-10 |
| mmu2404192 | **Signaling by Type 1 Insulin-like Growth Factor 1 Receptor (IGF1R)** | | 261 | 3.75 | 7,64E-10 |
| mmu2428928 | **IRS-related events triggered by IGF1R** | | 261 | 3.75 | 7,64E-10 |
| mmu2428924 | **IGF1R signaling cascade** | | 261 | 3.75 | 7,64E-10 |
| mmu187037 | **NGF signalling via TRKA from the plasma membrane** | | 312 | 3.43 | 1,41E-09 |
| mmu5654716 | **Downstream signaling of activated FGFR4** | | 281 | 3.56 | 2,16E-09 |
| mmu5654708 | **Downstream signaling of activated FGFR3** | | 281 | 3.56 | 2,16E-09 |
| mmu5654696 | **Downstream signaling of activated FGFR2** | | 281 | 3.56 | 2,16E-09 |
| mmu512988 | **Interleukin-3, 5 and GM-CSF signaling** | | 227 | 3.91 | 2,69E-09 |
| mmu5654743 | **Signaling by FGFR4** | | 284 | 3.52 | 3,05E-09 |
| mmu5654687 | **Downstream signaling of activated FGFR1** | | 284 | 3.52 | 3,05E-09 |
| mmu5654741 | **Signaling by FGFR3** | | 285 | 3.51 | 3,41E-09 |
| mmu166520 | **Signalling by NGF** | | 395 | 3.05 | 3,82E-09 |
| mmu5654736 | **Signaling by FGFR1** | | 289 | 3.46 | 5,34E-09 |
| mmu177929 | **Signaling by EGFR** | | 303 | 3.38 | 6,83E-09 |
| mmu169893 | **Prolonged ERK activation events** | | 216 | 3.90 | 1,08E-08 |
| mmu912526 | **Interleukin receptor SHC signaling** | | 216 | 3.90 | 1,08E-08 |
| mmu179812 | **GRB2 events in EGFR signaling** | | 208 | 3.94 | 1,56E-08 |
| mmu180336 | **SHC1 events in EGFR signaling** | | 208 | 3.94 | 1,56E-08 |
| mmu5654712 | **FRS-mediated FGFR4 signaling** | | 209 | 3.92 | 1,78E-08 |
| mmu5654706 | **FRS-mediated FGFR3 signaling** | | 209 | 3.92 | 1,78E-08 |
| mmu5654700 | **FRS-mediated FGFR2 signaling** | | 209 | 3.92 | 1,78E-08 |
| mmu5654693 | **FRS-mediated FGFR1 signaling** | | 209 | 3.92 | 1,78E-08 |
| mmu187687 | **Signalling to ERKs** | | 221 | 3.81 | 2,05E-08 |
| mmu451927 | **Interleukin-2 signaling** | | 223 | 3.77 | 2,63E-08 |
| mmu201681 | **TCF dependent signaling in response to WNT** | | 150 | 4.55 | 2,76E-08 |
| mmu5218921 | **VEGFR2 mediated cell proliferation** | | 217 | 3.77 | 4,94E-08 |
| mmu2262752 | **Cellular responses to stress** | | 277 | 3.20 | 8,04E-07 |
| mmu449147 | **Signaling by Interleukins** | | 361 | 2.77 | 4,88E-06 |
| mmu76002 | **Platelet activation, signaling and aggregation** | | 255 | 3.12 | 1,15E-05 |
| mmu109581 | **Apoptosis** | | 86 | 5.02 | 2,87E-05 |
| mmu1280215 | **Cytokine Signaling in Immune system** | | 484 | 2.26 | 4,54E-04 |
| mmu114608 | **Platelet degranulation** | | 130 | 3.32 | 1,23E-02 |
| mmu3000178 | **ECM proteoglycans** | | 39 | 5.83 | 1,84E-02 |
| mmu2029482 | **Regulation of actin dynamics for phagocytic cup formation** | | 52 | 4.81 | 3,98E-02 |

mmu : Mus musculus

Size = nb of genes contained in the KEGG gene sets

FE: Fold Enrichment
