# Supplementary material for: Leishmania proteophosphoglycans regurgitated from infected sand flies accelerate dermal wound repair and exacerbate leishmaniasis via insulin-like growth factor 1-dependent signalling
Source: PLoS Pathog. 2018 Jan 19;14(1):e1006794. doi: 10.1371/journal.ppat.1006794 (PMC5792026; doi:10.1371/journal.ppat.1006794)
Supplement: S3 Table — The genes used to validate gene expression in the microarray study (Fig 1E) are denoted with an asterisk. (DOCX) [file ppat.1006794.s003.docx]

**Supplementary Table 3, Giraud E et al.**

| Abbreviation | Primer sequences | | Tm (°C) |
| --- | --- | --- | --- |
|  | Forward | Reverse |  |
| Akt* | CCGCCTCTGCTTTGTCAT | CGTCCTTGATCCCCTCCT | 84.2 |
| Anxa1* | TGCCTCACAACCATCGTG | TCCGAACGGGAGACCATA | 81.4 |
| Arg1 | GCAGTTGGAAGCATCTC | GTTGCCCATGCAGATT | 81.7 |
| Arg2* | CAAAAGGCAGAGGCCA | TCACATCCACTGCTAGTC | 79.6 |
| Ass1* | CCAACATTGGCCAGAAGG | TGGGCAATCTCCACCTGT | 83.1 |
| Azin1* | TGATGAGCCAGCCTTCGT | CACAGGATGGACCCCAAA | 78.0 |
| Casp1 | CCTGGCAGGAATTCTGGA | GAGGGCAAGACGTGTACGA | 78.8 |
| Casp3* | AAAGGACGGGTCGTGGTT | CGTTGCCACCTTCCTGTT | 80.7 |
| Cat* | CACGCAAAAGGAGCAGGT | GGGGTGTTGTTTCCCACA | 81.3 |
| CCL2 | CAAGAAACGCAAGCGG | ACGGGATCTGAAAGACG | 84.0 |
| CCL3 | ACCACTGCCCTTGCTGTTC | TCTGCCGGTTTCTCTTAGTCAG | 82.6 |
| CCL4 | CCAATGGGCTCTGACC | ACTCCAAGTCACTCATGT | 81.7 |
| Cdc42* | GGAGTGCTCTGCCCTCAC | ACACACCTGCGGCTCTTC | 79.8 |
| Chuk* | GACGCCAGGGAGACTTGA | GCTTGCAGCCCAACAACT | 82.3 |
| Crk* | GACAGCCTTGGCTTTGGA | TGATCCAGCAGACGGACA | 80.2 |
| CXCL2 | CACCAACCACCAGGCTACAG | GCCCTTGAGAGTGGCTATGAC | 83.8 |
| Egf | CAAGCACGGCACAGTTTG | GGCATGGTCTTCCCCTCT | 82.0 |
| Egfr* | GCCAAACTGCTTGGTGCT | AAGGCTTGGACCCAAAGG | 79.1 |
| Fgfr2* | CTCACCCAGCCTCTCGAA | TAAGGCATGGGGTCTGGA | 79.0 |
| Fn1* | AGGCTGGATGATGGTGGA | CCGGCTGAAGCACTTTGT | 82.9 |
| Hmgb1* | ACCCGGATGCTTCTGTCA | TTTGATTTTGGGGCGGTA | 80.2 |
| Hsp90aa1* | GCCGATTTGGACGAGAAA | GAAGGGCCCGGAATTCTA | 77.2 |
| Igf1* | TGTTGCTTCCGGAGCTGT | TTGGGCATGTCAGTGTGG | 82.6 |
| Igf1r* | TGGAGGAGGTGACGGAAA | ATGGTCGTGTTGGCCACT | 82.0 |
| Ikkb* | ACGGAGGATGAGAGTC | TGAGATGCACTGAGTAGC | 82.6 |
| IL-1α* | CAACGTCAAGCAACGG | AATCACTCTGGTAGGTGT | 79.3 |
| IL-1β* | AGGCAGGCAGTATCAC | CACACCAGCAGGTTATC | 80.6 |
| IL-1Racp* | GACGCTGCGTGGAGTTTT | CCGGGAGGCCATATTTTC | 82.5 |
| IL-5* | AAACTGTCCGTGGGGGTA | AGCCTTCCATTGCCCACT | 82.6 |
| IL-6 | ACAACGATGATGCACTT | CTTGGTCCTTAGCCACT | 77.1 |
| IL-10 | CCAAGCCTTATCGGAAATG | CCTGAGGGTCTTCAGC | 79.3 |
| IL-18* | CAACCGCAGTAATACGGA | CAGGAGAGGGTAGACAT | 78.3 |
| IL-33* | ATTTCCCCGGCAAAGTTC | GGCCAGAACGGAGTCTCA | 81.0 |
| Inos2* | ACAGGAACCTACCAGC | GGTTGGACCACTGGAT | 81.9 |
| L19 | TACTGCCAATGCTCGG | AACACATTCCCTTTGACC | 80.9 |
| Lgals3* | AACCCAACGCAAACAGGA | GCTGATTTCCCGGAGGTT | 80.2 |
| Lgals9* | TGAACCCCCGTTTCAATG | TGGATATCACCCGCCACT | 82.1 |
| Mef2a* | TGCCGACAGCCTACAACA | CACGGGGAGGTGAAATTG | 84.5 |
| Myd88* | AGGCATCACCACCCTTGA | ACGGTCGGACACACACAA | 81.1 |
| Nono | AAAGCAGTTAGAACTCAGG | GGCCCATCCGTATCTC | 81.9 |
| Oaz1* | GCGGCTGAATGTGACAGA | AAACGTGGTCAGCCTGGA | 85.5 |
| Odc1* | AGAAGAGACCCAAGCC | GCAACAGTGTATGCACC | 80.9 |
| P38* | GTTCTACCGGCAGGAG | GCCCCGTCTTTGTATCA | 84.0 |
| Rhoa* | GGCTGCCATCAGGAAGAA | ACCTCGATATCCGCCACA | 80.3 |
| Slc25a15* | ACCATGGGCTCTCAAGCA | ACAGGCAAATCCCACCAA | 80.2 |
| SsrRNA | CCATGTCGGATTTGGT | CGAAACGGTAGCCTAGAG | 80.8 |
| Stat1 | TGGGTGCATTATGGGC | TTTCGTGTAGGGCTCC | 83.8 |
| Stat3 | TGCGGAGAAGCATTGTGA | ATTTGTTGGCGGGTCTGA | 83.9 |
| Stat6 | TTGCACACGTCATCCG | ACTGAGCAAGATCCCG | 81.4 |
|  |  |  |  |
| Tgf-β* | GCGGACTACTATGCTAAAGA | GTAACGCCAGGAATTGT | 76.5 |
| Tnfa | CATCAGTTCTATGGCCC | GTGAGGAGCACGTAGT | 85.1 |
| Ym1 | AGTTGGGCTAAGGACAG | ACGCAAGTCTTGCTCA | 78.9 |
| Ywhaz | GTTACTTGGCCGAGGT | GGAGTTCAGGATCTCGT | 81.0 |
