# Supplementary figures and images for: Leishmania proteophosphoglycans regurgitated from infected sand flies accelerate dermal wound repair and exacerbate leishmaniasis via insulin-like growth factor 1-dependent signalling
Source: PLoS Pathog. 2018 Jan 19;14(1):e1006794. doi: 10.1371/journal.ppat.1006794 (PMC5792026; doi:10.1371/journal.ppat.1006794)

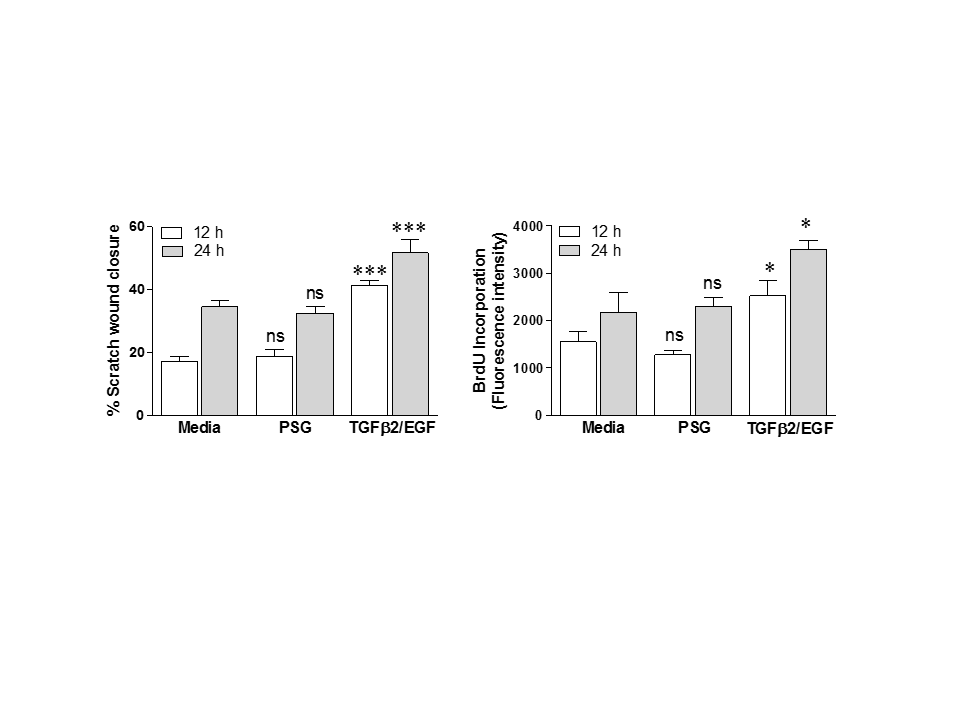

Supplement: S1 Fig — Monolayers of Kera308 keratinocytes were scratched in the presence of culture media supplemented with or without 0.5 μg/ml L. mexicana PSG. Positive controls were treated with 10 μg/ml TGFβ2 and 10 μg/ml EGF. A) At 0, 12 and 24 hours post-wound, photomicrographs were taken and scratch closure was determined from using ImageJ. Statistical analyses were performed between Media vs. PSG at each time point. Each in condition was performed in quadruplicate, data is pooled from 3 experiments. Average wound closure ±SEM is shown (*: p<0.05; **: p<0.005; ***: p<0.0005 by Mann Whitney t-test). (TIF) [file ppat.1006794.s004.TIF]

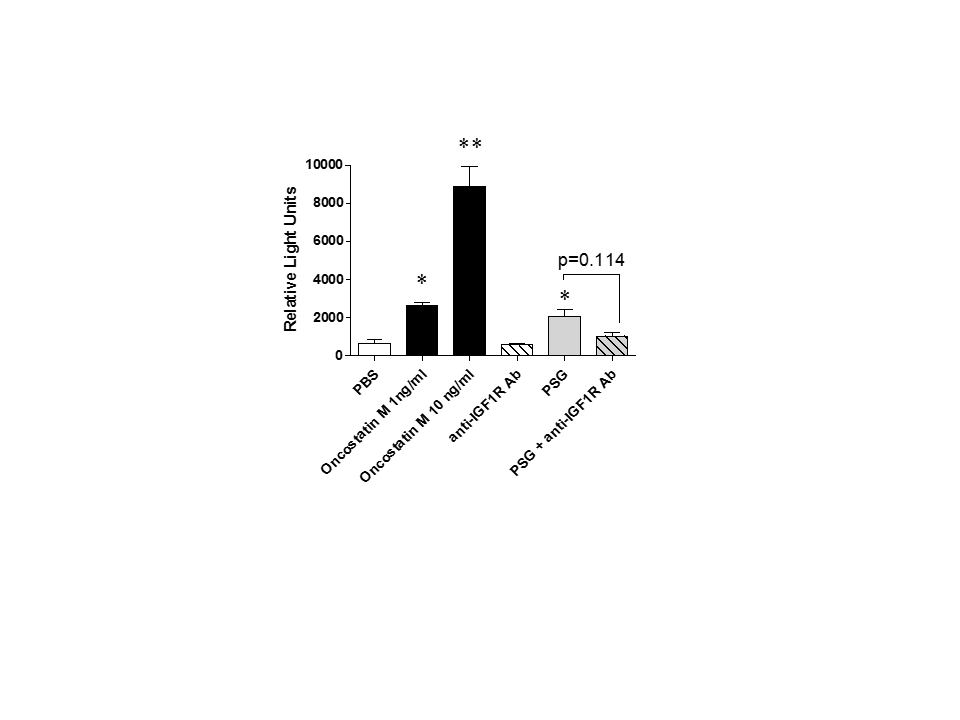

Supplement: S2 Fig — STAT3-Luciferase reporter HeLa cells were exposed to 1 μg PSG ± 1:100 anti-IGF1R Ab for 24 hours. Positive controls were treated with 1 ng/ml and 10 ng/ml Oncostatin M for 8 hours. Luciferase expression was recorded with a luminomter. Results are from a representative experiment. Relative light intensity is presented as the mean ±SD of 4 wells per group. (*: p<0.05, **: p<0.005 by Mann Whitney t-test). (TIF) [file ppat.1006794.s005.TIF]

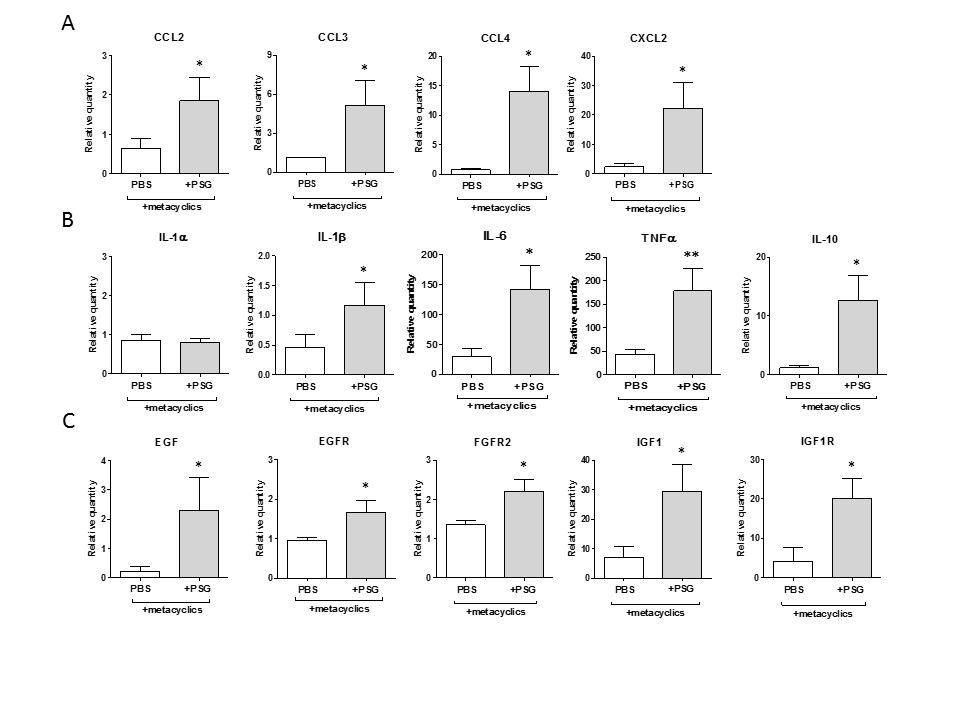

Supplement: S3 Fig — Ears of BALB/c mice were intra-dermally inoculated with 1 x 10^3^ L. mexicana metacyclic promastigotes ±0.5 μg L. mexicana PSG or PBS. A-C) Six hours post-infection ears were measured for transcripts involved in the inflammation and cell proliferation phases of wound healing by real-time quantitative PCR. A) Chemokines: CCL2, CCL3, CCL4 and CXCL2, B) pro-inflammatory-modulating cytokines: IL-1α, IL-1β, IL-6, IL-10 and TNFα, and C) epidermal growth factors and receptors: EGF, IGF1, EGFR, IGF1R and FGFR2. Relative expression was normalised to the housekeeping genes nono and l19 and is presented as the mean ±SD with 9–12 mice per group. (*: p<0.05, **: p<0.005 by Mann Whitney t-test). (TIF) [file ppat.1006794.s006.TIF]

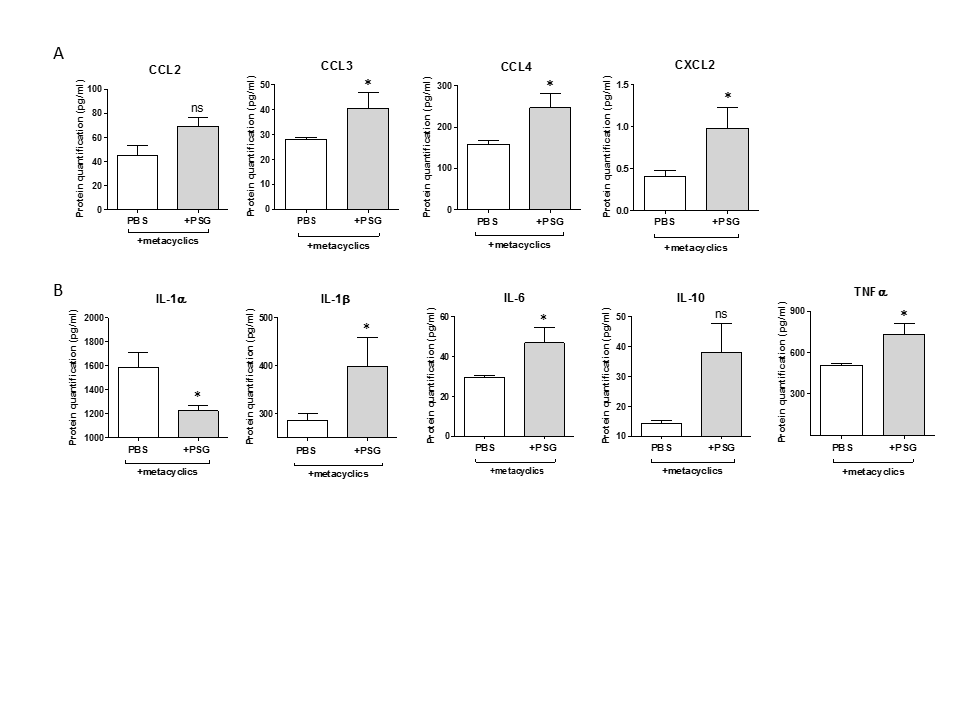

Supplement: S4 Fig — Ears of BALB/c mice were intra-dermally inoculated with 1 x 10^3^ L. mexicana metacyclic promastigotes ±0.5 μg L. mexicana PSG or PBS. A and B) Twenty four hours post-infection ears were measured for proteins involved in the inflammation phase of wound healing by real-time quantitative PCR. A) Chemokines: CCL2, CCL3, CCL4 and CXCL2 and B) pro-inflammatory-modulating cytokines: IL-1α, IL-1β, IL-6, IL-10 and TNFα. Protein levels were determined by Luminex from the ear lysates used in S3 Fig, and is presented as the mean ±SD with 9–12 mice per group. (*: p<0.05 by Mann Whitney t-test). (TIF) [file ppat.1006794.s007.TIF]

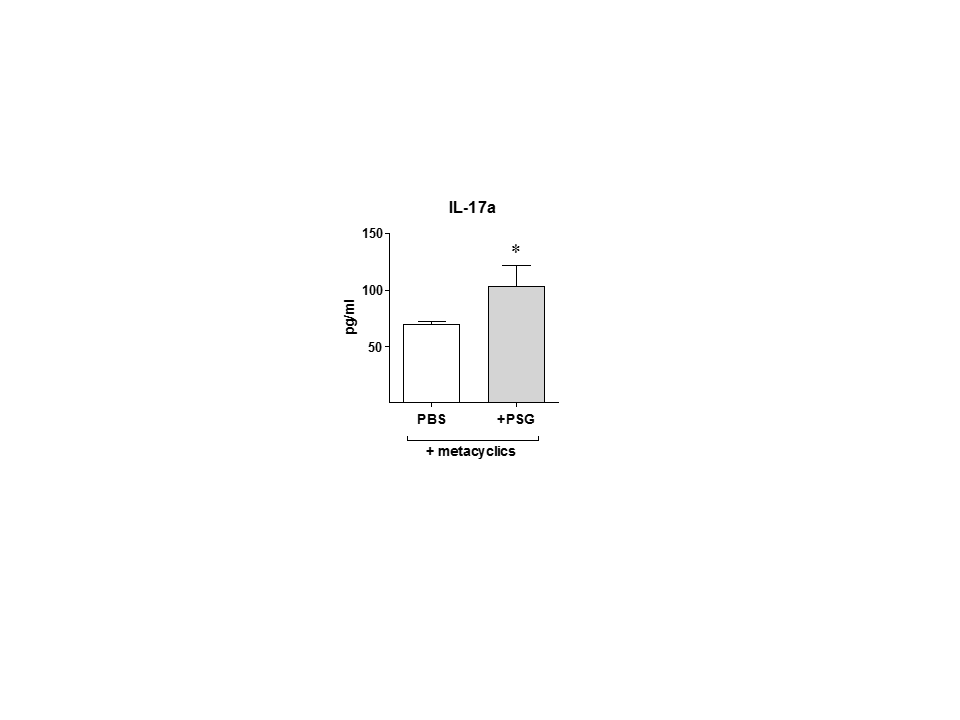

Supplement: S5 Fig — Ears of BALB/c mice were intra-dermally inoculated with 1 x 10^3^ L. mexicana metacyclic promastigotes ±0.5 μg/ml L. mexicana PSG. IL-17a production was determined from whole cell lysates using Luminex. Data is representative duplicate experiments (*: p<0.05 by Mann Whitney t-test). (TIF) [file ppat.1006794.s008.TIF]
